# Needs of parents and carers of children and young people with mental health difficulties: protocol for a systematic review

**DOI:** 10.1136/bmjopen-2022-071341

**Published:** 2023-02-16

**Authors:** Faith Martin, Dania Dahmash, Sarah Glover, Charlie Duncan, Andy Turner, Sarah L Halligan

**Affiliations:** 1 School of Psychology, Cardiff University, Cardiff, UK; 2 Centre for Intelligent Healthcare, Coventry University, Coventry, UK; 3 Parental Minds, Honiton, UK; 4 British Association for Counselling and Psychotherapy, Lutterworth, UK; 5 Department of Psychology, University of Bath, Bath, UK

**Keywords:** MENTAL HEALTH, Child & adolescent psychiatry, PUBLIC HEALTH

## Abstract

**Introduction:**

Having a child or young person (CYP) with mental health problems can be highly distressing for parents/carers. The impact can include parental/carer depression, anxiety, lost productivity and poor family relationships. Currently, there is no synthesis of this evidence, which is needed to provide clarity around what support parents/carers may need, to meet the needs of family mental health. This review aims to identify the needs of the parents/carers of CYP who are receiving mental health services.

**Methods and analysis:**

A systematic review will be conducted to identify potentially relevant studies that provide evidence concerning the needs and impact on parents/carers linked to their CYP having mental health difficulties. CYP mental health conditions included are anxiety disorders, depression, psychoses, oppositional defiant and other externalising disorders, labels of emerging personality disorders, eating disorders and attention deficit (hyperactive) disorders. The following databases were searched on November 2022 with no date restriction applied: Medline; PsycINFO; CINAHL; AMED; EMBASE; Web of Science; Cochrane Library; WHO International Clinical Trials Registry Platform; Social Policy and Practice; Applied Social Sciences Index and Abstracts; and Open Grey. Only studies reported in English will be included. The quality of the included studies will be assessed using Joanna Briggs Institute Critical Appraisal Checklist for qualitative studies and the Newcastle Ottawa Scale for quantitative studies. Qualitative data will be analysed thematically and inductively.

**Ethics and dissemination:**

This review was approved by the ethical committee at Coventry University, UK, reference number P139611. The findings from this systematic review will be disseminated across various key stakeholders and published in peer-reviewed journals.

**STRENGTHS AND LIMITATIONS OF THIS STUDY**

- This systematic review protocol follows the Preferred Reporting Items for Systematic Review and Meta-Analysis Protocols guidelines.
- Qualitative and quantitative studies will be included to provide a comprehensive review of the impact on and needs of parents/carers of young people with mental health difficulties.
- The review will be limited to studies published in English, raising a potential language bias.

## Introduction

Almost 1 250 000, aged 5–19 years old in the UK alone, have a diagnosable mental illness and statutorily funded UK Child and Adolescent Mental Health Services (CAMHS) have capacity to see just one-third of these.^1^ Before COVID-19, the prevalence of mental health (MH) disorders among 5–16 years old was 10%, which has increased to an estimated 16% post-COVID-19.^2^ This means greater demand on already stretched CAMHS, and more parents/carers trying to provide support.

There is a close association between children and young people’s (CYP’s) MH and that of their parents/carers.^3^ Rates of diagnosed depression and other MH problems are higher among parents/carers for CYP with MH problems compared with other parents: 51% compared with 23%.^4–6^ Having a child with an MH problem can be distressing for parents/carers, impacting their well-being, stress and relationship with their CYP.^7 8^ This leads to greater service use by parents/carers for their own psychological well-being and lost productivity through time off work. It is critical that we support parents/carers, in relation to their individual needs, the impact on the wider family and for the indirect effect of the CYP.

Unfortunately, parents/carers are typically left needing information, support in helping their young people and emotional support for themselves with the impact of seeing their young person in distress.^8–10^ The impact of CYP MH on parents/carers is acknowledged, for example, the recent UK Care Quality Commission report suggests parents/carers be seen as partners in CYP MH care.^11^ There is then growing policy attention on supporting parents/carers, but a lack of consideration of the parents’/carers’ own needs and well-being, as part of the family system. Improving CAMHS with an ‘integrated whole family approach’ and greater support for parents/carers in supporting their CYP have been proposed.^6 12^ To achieve this, an understanding of the impact of CYP MH problems on parents/carers is essential, to inform interventions. Currently a synthesised understanding is absent from treatment and clinical guidelines and there are no literature reviews that provide guidance on how to support these parents/carers. This proposal was designed to meet this critical need.

Hence, this systematic review aims to synthesise current evidence in relation to parents’/carers’ needs of young people who are receiving mental services, in order to then develop and improve interventions to support these parents/carers.

## Method and analysis

This systematic review has been approved by the ethical committee at Coventry University, UK (reference number P139611). This protocol follows the Preferred Reporting Items for Systematic Review and Meta-Analysis for Protocols (PRISMA-P) 2015 guidelines.^13^

The age range of CYP is defined as 5–18 to reflect the upper limit of most commissioned CAMHS services in the UK. The lowest limit reflects the lower limit of the age of onset of most conditions^14^ and the lower bound of UK surveillance data.^15^ Studies where the majority (over 50%) of the sample are parents/carers of CYP aged 5–18 will be included.

The term ‘parents/carers’ includes biological and step-parents, other relatives assuming a parenting role, non-biological and adoptive parents, foster carers, and other adults in legal guardian roles. The CYP may have any MH condition, including depression, anxiety disorders, psychoses, oppositional defiant and other externalising disorders, labels of emerging personality disorders, eating disorders and attention deficit (hyperactive) disorders. This review does not cover issues in relation to the primary prevention of MH difficulties in childhood. The review does not cover parents/carers of children solely with special educational needs, including autism spectrum conditions or developmental language disorders. Studies with samples exclusively focused on parents/carers of CYP with these conditions will be excluded. Studies that focus on CYP with MH difficulties and within that include less than 50% CYP with developmental/special educational needs would be included.

### Eligibility criteria

To be included in this review, studies must include participants who are parents/carers of CYP (aged 5–18), where the CYP has an MH difficulty (as detailed above). The CYP must have received a formal diagnosis and/or have accessed MH services, be that via community services, primary care MH services, secondary, inpatient, specialist services or via healthcare professionals in schools. Studies must provide primary quantitative or qualitative data relating to parents’/carers’ needs or impact on parents/carers from CYPs MH difficulty. Outcomes or observations must relate to the impact of having a CYP with an MH problem on the parents/carers and the needs they have in relation to that impact. Outcomes in quantitative studies must use validated measures only. Information and knowledge needs; parents’/carers’ own MH needs in relation to depression, anxiety and stress; and parents’/carers’ difficulties with poor parenting satisfaction, family relationship problems and low parenting self-efficacy will be included. Only studies published in English will be included due to limited resources. No date restriction will be applied. A PRISMA flow chart will detail the complete study selection process.

### Study designs

Studies of any design, other than systematic reviews, are eligible for inclusion.

### Interventions and outcomes

This review focuses on understanding the impact on parents/carers of having a CYP with an MH difficulty, and research that seeks to specify their needs. As such, studies may include interventions, however, for this review, we will only extract data where this describes need/impact. Quantitative studies must use validated measures, and report either comparison to a control group or use measures that have well established norms/clinical cut-off scores. This is to allow us to interpret the extent of the impact or need in our target population. Relevant outcomes are parental/carer stress, burn-out, depression, anxiety and stress; and parents’/carers’ parenting satisfaction, family relationships and parenting self-efficacy.

### Search strategy

The following bibliographic databases were searched from inception to November 2022: Medline; PsycINFO; CINAHL; AMED; EMBASE; Web of Science; Cochrane Library (including Cochrane Database of Systematic Reviews, Cochrane Central Register of Controlled Trials, Database of Abstracts of Reviews of Effects, Health Technology Assessment Database, and National Health Service (NHS) Economic Evaluation Database); WHO International Clinical Trials Registry Platform (searches multiple databases including clinicaltrials.gov); Social Policy and Practice; Applied Social Sciences Index and Abstracts; and Open Grey. The search strategy for Medline is shown in table 1, and the full search strategies for all databases are shown in online supplemental file 1.

10.1136/bmjopen-2022-071341.supp1 Supplementary data

**Table 1:** Search strategy for Medline

Search	Terms
S1 (Parent)	TI ((parent or parents or parental or mother or father or care*giver or guardian* or carer* or paternal or maternal)) OR AB ((parent or parents or parental or mother or father or care*giver or guardian* or carer* or paternal or maternal)) OR MM (“Parents+")
S2 (Children)	TI ((children or adolescent* or adolescence or youth* or child or teenager* or pediatric* or paediatric* or kid* or teen* or young person or young people or boy* or girl* or juvenile*)) OR AB ((children or adolescent* or adolescence or youth* or child or teenager* or pediatric* or paediatric* or kid* or teen* or young person or young people or boy* or girl* or juvenile*)) OR MH (“Child+“) OR MM (“Adolescent”)
S3 (Mental Health)	(TI ((attention deficit disorder* or “attention deficit hyperactive disorder* “or “ADHD”) OR AB (attention deficit disorder* or “attention deficit hyperactive disorder* “or “ADHD”) OR MH (“Attention Deficit Disorder with Hyperactivity”) OR (TI ((Eating disorder* or eating problem*)) OR AB ((Eating disorder* or eating problem*) OR MH(“Feeding and Eating Disorders+”) OR (TI ((Emerging personality disorder* or emerging personality problem*)) OR AB ((Emerging personality disorder* or emerging personality problem*)) OR MH (“Personality Disorder+”)) OR (TI ((Externalising disorder* or externalising problem* or externalizing disorder* or externalizing problem*) OR AB (Externalising disorder* or externalising problem* or externalizing disorder* or externalizing problem*) OR TX (“Externalising disorder”)) OR (TI ((Oppositional defiant disorder* or oppositional defiant problem*)) OR AB ((Oppositional defiant disorder* or oppositional defiant problem*) OR (MH“Attention Deficit and Disruptive Behavior Disorders+”)) OR (TI ((Psychos* or psychotic disorder* or psychotic problem*)) OR AB ((Psychos* or psychotic disorder* or psychotic problem*)) OR MH (“Psychotic Disorders+)) OR (TI ((Anxiety or depression or depressive or “obsessive compulsive disorder” or “OCD” or phobia or phobic or mood disorder or anxiety disorder or panic disorder or agoraphobia or internalising problem* or internalising problem* or internalizing problem* or internalizing disorder*)) OR AB ((Anxiety or depression or depressive or “obsessive compulsive disorder” or “OCD” or phobia or phobic or mood disorder or anxiety disorder or panic disorder or agoraphobia or internalising problem* or internalising problem* or internalizing problem* or internalizing disorder*) OR (MH “Depressive Disorder”) OR (MH “Depressive Disorder, Major”) OR (MH “Depressive Disorder, Treatment-Resistant”) OR (MH “Dysthymic Disorder”) OR (MM “Anxiety Disorders+"))
S4 (Needs or impact)	TI (“Information need*” or “knowledge need*” or need* or support or experience*or impact or wellbeing or concern* or want or perspective* or belief* or attitude*or prefer* or anxiety or anxious or depressed or depression or strain or stress or burden or “parent satisfaction” or “family relationship” or “parent* self-efficacy”) OR AB (“Information need*” or “knowledge need*” or need* or support or experience*or impact or wellbeing or concern* or want or perspective* or belief* or attitude*or prefer* or anxiety or anxious or depressed or depression or strain or stress or burden or “parent satisfaction” or “family relationship” or “parent* self-efficacy”))
S5	S1 N8 S4
S6	S2 N8 S3

The reference lists of relevant systematic reviews will be screened for additional studies not captured by the database search. Cited reference searches and examination of reference lists will be conducted for related systematic reviews and any key papers identified.

The search terms will include four blocks of terms (table 1), all of which must be present, each relating to a key element of the review scope:

1. Parents or carers.
2. Children and young people.
3. MH problems.
4. Needs or impact.

### Selection process

Rayyan software will be used to manage citations.^16^ It facilitates blind scoring for inclusion/exclusion. Inclusion/exclusion criteria will undergo a final review by a patient public involvement (PPI) group.

A two-step process will be used for screening. First, all titles and abstracts will be screened against the inclusion/exclusion criteria by two people independently. Second, full texts will be obtained for short-listed studies and formal inclusion/exclusion will be conducted by two reviewers independently. Any discrepancies will be resolved by a third reviewer if needed.

### Data extraction and quality appraisal

Data will be extracted into an Excel sheet following Cochrane guidelines and Joanna Briggs Institute (JBI).^17 18^ The data extraction sheet will be piloted using papers relevant to the research question and refined. Data extracted using the pilot form will then be rechecked using the final form.

All study characteristics (population, intervention and comparator details) will be extracted, as well as study design, sample size and sample characteristics (including parent/carer and their CYP characteristics). Quantitative data that captures the needs or impact of parents/carers relating to their CYP’s MH problems will be extracted. Quantitative studies with a single group of participants (parents/carers exposed to CYP self-harm) must use validated measures with established norms or clinical cut-offs, to allow interpretation of findings. Quantitative designs such as case–control, where a comparison group of parents/carers not exposed to CYP self-harm must use any validated measure, as levels of well-being etc in our target group can be interpreted in relation to the control group. Qualitative data describing parents’/carers’ needs or their impact on them will be extracted in detail. Data extraction will include mean scores, SD, percentage of participants in the ‘clinical’ cut-off range and effect sizes for case–control designs.

For the quality appraisal, the following tools will be used: JBICritical Appraisal Checklist^17^ for the qualitative studies and the Newcastle Ottawa Scale^19^ for quantitative studies.

### Data synthesis

Qualitative descriptions of impact or need will be synthesised thematically and inductively.^17 20^ Two reviewers will do this independently, then discuss together, with decisions made with other reviewers and stakeholder input where needed.^21^ The impact on parents/carers and their needs will be visually mapped to capture any links between these. We will also group findings in relation to CYP diagnosis and examine differences relating to the gender/identity of parent/carer.

Quantitative reports will be grouped by variable measures (eg, depression, satisfaction with parenting, information needs) and summarised, with critical reflection on methodological quality. We anticipate inconsistency between studies in relation to measures used and comparison groups, rendering quantitative data synthesis unlikely.

A summary list of needs/impacts will be created based on these findings. The stakeholders, including our parent/carer working group, will provide input on the themes, proposed links between them and any needs/impacts/populations they perceive as under-researched.

The quantitative data will be integrated with qualitative findings by using the convergent integrated approach of mixed methods reviews proposed by JBI. This method is appropriate as the research questions can be answered by both quantitative and qualitative synthesis. The synthesis will be achieved by qualitising quantitative findings into textural statements and pooling them with qualitative data extracted directly from qualitative studies. Repeated, a detailed examination of the assembled data will be undertaken to identify categories based on similarity in meaning. This will determine if the results confirm/expand/refute one another. A category will integrate two or more qualitative data, qualitised data or a combination of both. Where possible, categories will be aggregated to produce overall finding(s). Where data cannot be categorised, findings will be reported narratively.

### Patient and public involvement

The scope of the review has been defined through discussion with Emerging Minds funded Special Interest Research Group on ‘Parents Support and Well-being’, which includes psychologists, academics, parents/carers and charity representatives. In addition, we consulted with parents/carers who are members of parent support groups (eg, coauthor SG’s group ‘arental Minds’) and more widely across the UK’s national network of parents’ support groups (the ‘PLACE’ network, hosted by our collaborators Charlie Waller Memorial Trust). The PPI group helped to design the study and specify the protocol, including the exclusion of autistic spectrum disorders and development difficulties from this review.

The PPI group will also be involved in the design of outputs and dissemination activities, including reviewing our interpretations for the formal reports and cowriting the outputs for a non-academic audience.

## Ethics and dissemination

This study was approved by Coventry University ethical committee, reference number P139611.

Dissemination will use multiple channels, including a project website, hosted by Coventry University. We will press release and widely disseminated through social media channels and YouTube, with support from the university’s marketing department and our collaborating organisations’ press offices.

We will disseminate findings via all our collaborators. Charlie Waller Trust’s national network of parent support organisations and wider links to MH providers and trainers. Young Minds sit on several national MH advisory groups, will organise a government round table dissemination event, and publicise our findings across their widely accessed communication channels. The Emerging Minds Network also have a large audience of public, academics and policy organisations.

We will disseminate via the Future NHS Collaboration platform, which reaches clinicians, service leads, planners, commissioners and NHS England.

The Local Government Association has created the Mental Health Challenge (www.mentalhealthchallenge.org.uk) to lead and direct local council policy on MH. We will use these channels to share our findings.

Parents/carers involved in the project will be sent the final outputs via email links/hard-copy. In addition, findings from this review will be published in a peer reviewed journal.

## Supplementary Material

Reviewer comments

Author's manuscript
