# Supplementary material for: Needs of parents and carers of children and young people with mental health difficulties: protocol for a systematic review
Source: BMJ Open. 2023 Feb 16;13(2):e071341. doi: 10.1136/bmjopen-2022-071341 (PMC9936057; doi:10.1136/bmjopen-2022-071341)
Supplement: Supplementary data [file bmjopen-2022-071341supp001.pdf]

Search conducted 8th November 2022 or thereabouts

Database: Medline

Date: 8th November 2022

**Search**	**Terms**
S1 (Parent)	TI ((parent or parents or parental or mother or father or care*giver or guardian* or carer* or paternal or maternal)) OR AB ((parent or parents or parental or mother or father or care*giver or guardian* or carer* or paternal or maternal)) OR MM ("Parents+")
S2 (Children)	TI ((children or adolescent* or adolescence or youth* or child or teenager* or pediatric* or paediatric* or kid* or teen* or young person or young people or boy* or girl* or juvenile*)) OR AB ((children or adolescent* or adolescence or youth* or child or teenager* or pediatric* or paediatric* or kid* or teen* or young person or young people or boy* or girl* or juvenile*)) OR MH ("Child+") OR MM ("Adolescent")
S3 (Mental Health)	(TI ((attention deficit disorder* or “attention deficit hyperactive disorder* “or “ADHD”) OR AB (attention deficit disorder* or “attention deficit hyperactive disorder* “or “ADHD”) OR MH (“Attention Deficit Disorder with Hyperactivity”) OR (TI ((Eating disorder* or eating problem*)) OR AB ((Eating disorder* or eating problem*) OR MH(“Feeding and Eating Disorders+”) OR (TI ((Emerging personality disorder* or emerging personality problem*)) OR AB ((Emerging personality disorder* or emerging personality problem*)) OR MH (“Personality Disorder+”)) OR (TI ((Externalising disorder* or externalising problem* or externalizing disorder* or externalizing problem*) OR AB (Externalising disorder* or externalising problem* or externalizing disorder* or externalizing problem*) OR TX (“Externalising disorder”)) OR (TI ((Oppositional defiant disorder* or oppositional defiant problem*)) OR AB ((Oppositional defiant disorder* or oppositional defiant problem*) OR (MH“Attention Deficit and Disruptive Behavior Disorders+”)) OR (TI ((Psychos* or psychotic disorder* or psychotic problem*)) OR AB ((Psychos* or psychotic disorder* or psychotic problem*)) OR MH (“Psychotic Disorders+)) OR (TI ((Anxiety or depression or depressive or “obsessive compulsive disorder” or “OCD” or phobia or phobic or mood disorder or anxiety disorder or panic disorder or agoraphobia or internalising problem* or internalising problem* or internalizing problem* or internalizing disorder*)) OR AB ((Anxiety or depression or depressive or “obsessive compulsive disorder” or “OCD” or phobia or phobic or mood disorder or anxiety disorder or panic disorder or agoraphobia or internalising problem* or internalising problem* or internalizing problem* or internalizing disorder*) OR (MH "Depressive Disorder") OR (MH "Depressive Disorder, Major") OR (MH "Depressive Disorder, Treatment-Resistant") OR (MH "Dysthymic Disorder") OR (MM "Anxiety Disorders+"))
S4 (Needs)	TI (“Information need*” or “knowledge need*” or need* or support or experience*or impact or wellbeing or concern* or want or perspective* or belief* or attitude*or prefer* or anxiety or anxious or depressed or depression or strain or stress or burden or “parent satisfaction” or “family relationship” or “parent* self-efficacy”) OR AB (“Information need*” or “knowledge need*” or need* or support or experience*or impact or wellbeing or concern* or want or perspective* or belief* or attitude*or prefer* or anxiety or anxious or depressed or depression or strain or stress or burden or “parent satisfaction” or “family relationship” or “parent* self-efficacy”))
S5	S1 N8 S4
S6	S2 N8 S3
S7	S5 AND S6

Database: PsycINFO

Date: 8th November 2022

Search	Terms
S1 (Parent)	TI ((parent or parents or parental or mother or father or care*giver or guardian* or carer* or paternal or maternal)) OR AB ((parent or parents or parental or mother or father or care*giver or guardian* or carer* or paternal or maternal)) OR MM ("Parents+")
S2 (Children)	TI ((children or adolescent* or adolescence or youth* or child or teenager* or pediatric* or paediatric* or kid* or teen* or young person or young people or boy* or girl* or juvenile*)) OR AB ((children or adolescent* or adolescence or youth* or child or teenager* or pediatric* or paediatric* or kid* or teen* or young person or young people or boy* or girl* or juvenile*)) OR MH ("Child+") OR MM ("Adolescent")
S3 (Mental Health)	(TI ((attention deficit disorder* or "attention deficit hyperactive disorder* "or "ADHD") OR AB (attention deficit disorder* or "attention deficit hyperactive disorder* "or "ADHD")OR MH ("Attention Deficit Disorder with Hyperactivity") OR (TI ((Eating disorder* or eating problem*)) OR AB ((Eating disorder* or eating problem*) OR MH("Feeding and Eating Disorders+") OR (TI ((Emerging personality disorder* or emerging personality problem*)) OR AB ((Emerging personality disorder* or emerging personality problem*)) OR MH ("Personality Disorder+")) OR (TI ((Externalising disorder* or externalising problem* or externalizing disorder* or externalizing problem*) OR AB (Externalising disorder* or externalising problem* or externalizing disorder* or externalizing problem*) OR TX ("Externalising disorder")) OR (TI ((Oppositional defiant disorder* or oppositional defiant problem*)) OR AB ((Oppositional defiant disorder* or oppositional defiant problem*) OR (MH"Attention Deficit and Disruptive Behavior Disorders+")) OR (TI ((Psychos* or psychotic disorder* or psychotic problem*)) OR AB ((Psychos* or psychotic disorder* or psychotic problem*)) OR MH ("Psychotic Disorders+)) OR (TI ((Anxiety or depression or depressive or "obsessive compulsive disorder" or "OCD" or phobia or phobic or mood disorder or anxiety disorder or panic disorder or agoraphobia or internalising problem* or internalising problem* or internalizing problem* or internalizing disorder*)) OR AB ((Anxiety or depression or depressive or "obsessive compulsive disorder" or "OCD" or phobia or phobic or mood disorder or anxiety disorder or panic disorder or agoraphobia or internalising problem* or internalising problem* or internalizing problem* or internalizing disorder*) OR (MH "Depressive Disorder") OR (MH "Depressive Disorder, Major") OR (MH "Depressive Disorder, Treatment-Resistant") OR (MH "Dysthymic Disorder") OR (MM "Anxiety Disorders+"))
S4 (Needs)	TI ("Information need*" or "knowledge need*" or need* or support or experience*or impact or wellbeing or concern* or want or perspective* or belief* or attitude*or prefer* or anxiety or anxious or depressed or depression or strain or stress or burden or "parent satisfaction" or "family relationship" or "parent* self-efficacy") OR AB ("Information need*" or "knowledge need*" or need* or support or experience*or

	impact or wellbeing or concern* or want or perspective* or belief* or attitude*or prefer* or anxiety or anxious or depressed or depression or strain or stress or burden or “parent satisfaction” or “family relationship” or “parent* self-efficacy”))
S5	S1 N8 S4
S6	S2 N8 S3
S7	S5 AND S6
S8	S5 AND S6 (English only)

Database: CINAHL ULTIMATE

Date: 8th November 2022

**Search**	**Terms**
S1 (Parent)	TI ((parent or parents or parental or mother or father or care*giver or guardian* or carer* or paternal or maternal)) OR AB ((parent or parents or parental or mother or father or care*giver or guardian* or carer* or paternal or maternal)) OR MM ("Parents+")
S2 (Children)	TI ((children or adolescent* or adolescence or youth* or child or teenager* or pediatric* or paediatric* or kid* or teen* or young person or young people or boy* or girl* or juvenile*)) OR AB ((children or adolescent* or adolescence or youth* or child or teenager* or pediatric* or paediatric* or kid* or teen* or young person or young people or boy* or girl* or juvenile*)) OR MH ("Child+") OR MM ("Adolescent")
S3 (Mental Health)	(TI ((attention deficit disorder* or “attention deficit hyperactive disorder* “or “ADHD”) OR AB (attention deficit disorder* or “attention deficit hyperactive disorder* “or “ADHD”)OR MH (“Attention Deficit Disorder with Hyperactivity”) OR (TI ((Eating disorder* or eating problem*)) OR AB ((Eating disorder* or eating problem*) OR MH(“Feeding and Eating Disorders+”) OR (TI ((Emerging personality disorder* or emerging personality problem*)) OR AB ((Emerging personality disorder* or emerging personality problem*)) OR MH (“Personality Disorder+”)) OR (TI ((Externalising disorder* or externalising problem* or externalizing disorder* or externalizing problem*) OR AB (Externalising disorder* or externalising problem* or externalizing disorder* or externalizing problem*) OR TX (“Externalising disorder”)) OR (TI ((Oppositional defiant disorder* or oppositional defiant problem*)) OR AB ((Oppositional defiant disorder* or oppositional defiant problem*) OR (MH“Attention Deficit and Disruptive Behavior Disorders+”)) OR (TI ((Psychos* or psychotic disorder* or psychotic problem*)) OR AB ((Psychos* or psychotic disorder* or psychotic problem*)) OR MH (“Psychotic Disorders+)) OR (TI ((Anxiety or depression or depressive or “obsessive compulsive disorder” or “OCD” or phobia or phobic or mood disorder or anxiety disorder or panic disorder or agoraphobia or internalising problem* or internalising problem* or internalizing problem* or internalizing disorder*)) OR AB ((Anxiety or depression or depressive or “obsessive compulsive disorder” or “OCD” or phobia or phobic or mood disorder or anxiety disorder or panic disorder or agoraphobia or internalising problem* or internalising problem* or internalizing problem* or internalizing disorder*) OR (MH "Depressive Disorder") OR (MH "Depressive Disorder, Major") OR (MH "Depressive Disorder, Treatment-Resistant") OR (MH "Dysthymic Disorder") OR (MM "Anxiety Disorders+"))
S4 (Needs)	TI (“Information need*” or “knowledge need*” or need* or support or experience*or impact or wellbeing or concern* or want or perspective* or belief* or

	attitude*or prefer* or anxiety or anxious or depressed or depression or strain or stress or burden or "parent satisfaction" or "family relationship" or "parent* self-efficacy") OR AB ("Information need*" or "knowledge need*" or need* or support or experience*or impact or wellbeing or concern* or want or perspective* or belief* or attitude*or prefer* or anxiety or anxious or depressed or depression or strain or stress or burden or "parent satisfaction" or "family relationship" or "parent* self-efficacy"))
S5	S1 N8 S4
S6	S2 N8 S3
S7	S5 AND S6
S8	S5 AND S6 (English only)

Database: AMED

Date: 8th November 2022

**Search**	**Terms**
S1 (Parent)	TI ((parent or parents or parental or mother or father or care*giver or guardian* or carer* or paternal or maternal)) OR AB ((parent or parents or parental or mother or father or care*giver or guardian* or carer* or paternal or maternal)) OR MM ("Parents+")
S2 (Children)	TI ((children or adolescent* or adolescence or youth* or child or teenager* or pediatric* or paediatric* or kid* or teen* or young person or young people or boy* or girl* or juvenile*)) OR AB ((children or adolescent* or adolescence or youth* or child or teenager* or pediatric* or paediatric* or kid* or teen* or young person or young people or boy* or girl* or juvenile*)) OR MH ("Child+") OR MM ("Adolescent")
S3 (Mental Health)	(TI ((attention deficit disorder* or "attention deficit hyperactive disorder* "or "ADHD") OR AB (attention deficit disorder* or "attention deficit hyperactive disorder* "or "ADHD")OR MH ("Attention Deficit Disorder with Hyperactivity") OR (TI ((Eating disorder* or eating problem*)) OR AB ((Eating disorder* or eating problem*) OR MH("Feeding and Eating Disorders+") OR (TI ((Emerging personality disorder* or emerging personality problem*)) OR AB ((Emerging personality disorder* or emerging personality problem*)) OR MH ("Personality Disorder+")) OR (TI ((Externalising disorder* or externalising problem* or externalizing disorder* or externalizing problem*) OR AB (Externalising disorder* or externalising problem* or externalizing disorder* or externalizing problem*) OR TX ("Externalising disorder")) OR (TI ((Oppositional defiant disorder* or oppositional defiant problem*)) OR AB ((Oppositional defiant disorder* or oppositional defiant problem*) OR (MH"Attention Deficit and Disruptive Behavior Disorders+")) OR (TI ((Psychos* or psychotic disorder* or psychotic problem*)) OR AB ((Psychos* or psychotic disorder* or psychotic problem*)) OR MH ("Psychotic Disorders+)) OR (TI ((Anxiety or depression or depressive or "obsessive compulsive disorder" or "OCD" or phobia or phobic or mood disorder or anxiety disorder or panic disorder or agoraphobia or internalising problem* or internalising problem* or internalizing problem* or internalizing disorder*)) OR AB ((Anxiety or depression or depressive or "obsessive compulsive disorder" or "OCD" or phobia or phobic or mood disorder or anxiety disorder or panic disorder or agoraphobia or internalising problem* or internalising problem* or internalizing problem* or internalizing disorder*) OR (MH "Depressive Disorder") OR (MH "Depressive Disorder, Major") OR (MH "Depressive Disorder, Treatment-Resistant") OR (MH "Dysthymic Disorder") OR (MM "Anxiety Disorders+"))

S4 (Needs)	TI ("Information need*" or "knowledge need*" or need* or support or experience*or impact or wellbeing or concern* or want or perspective* or belief* or attitude*or prefer* or anxiety or anxious or depressed or depression or strain or stress or burden or "parent satisfaction" or "family relationship" or "parent* self-efficacy") OR AB ("Information need*" or "knowledge need*" or need* or support or experience*or impact or wellbeing or concern* or want or perspective* or belief* or attitude*or prefer* or anxiety or anxious or depressed or depression or strain or stress or burden or "parent satisfaction" or "family relationship" or "parent* self-efficacy"))
S5	S1 N8 S4
S6	S2 N8 S3
S7	S5 AND S6
S8	S5 AND S6 (English only)

Database: The Cochrane Library (including the Cochrane Database of Systematic Reviews, the Cochrane Central Register of Controlled Trials (CENTRAL), the Database of Abstracts of Reviews of Effects, the Health Technology Assessment Database, and the NHS Economic Evaluation Database)

Question number: 1

Date: 8th November 2022

Search	Terms
S1 (Parent)	TI ((parent or parents or parental or mother or father or care*giver or guardian* or carer* or paternal or maternal)) OR AB ((parent or parents or parental or mother or ather or care*giver or guardian* or carer* or paternal or maternal)) OR MM ("Parents+")
S2 (Children)	TI ((children or adolescent* or adolescence or youth* or child or teenager* or pediatric* or paediatric* or kid* or teen* or young person or young people or boy* or girl* or juvenile*)) OR AB ((children or adolescent* or adolescence or youth* or child or teenager* or pediatric* or paediatric* or kid* or teen* or young person or young people or boy* or girl* or juvenile*)) OR MH ("Child+") OR MM ("Adolescent")
S3 (Mental Health)*	attention deficit disorder* or attention deficit hyperactive disorder* or ADHD or Eating disorder* or eating problem* or Emerging personality disorder or emerging personality problem* or Externalising disorder* or externalising problem* or externalizing disorder* or externalizing problem* or Oppositional defiant disorder* or oppositional defiant problem* or Psychos* or psychotic disorder* or psychotic problem* or Anxiety or depression or depressive or obsessive compulsive disorder or OCD or phobia or phobic or mood disorder or anxiety disorder or panic disorder or agoraphobia or internalising problem* or internalising problem* or internalizing problem* or internalizing disorder*
S4 (Needs)	TI ("Information need*" or "knowledge need*" or need* or support or experience*or impact or wellbeing or concern* or want or perspective* or belief* or attitude*or prefer*) OR AB ("Information need*" or "knowledge need*" or need* or support or experience*or impact or wellbeing or concern* or want or perspective* or belief* or attitude*or prefer*) or (Anxiety) or (depression) or (stress) or (burnout) or (parent satisfaction) or (family relationships) or (parenting self-efficacy)
S5	S1 AND S2 AND S3 AND S4

Database: EMBASE

Date: 8th November 2022

Search	Terms
S1 (Parent)	(parent or parents or parental or mother or father or care*giver or guardian* or carer* or paternal or maternal).ab. or (parent or parents or parental or mother or father or care*giver or guardian* or carer* or paternal or maternal).ti. or parents.kw.
S2 (Children)	(children or adolescent* or adolescence or youth* or child or teenager* or pediatric* or paediatric* or kid* or teen* or young person or young people or boy* or girl* or juvenile*).ab. or (children or adolescent* or adolescence or youth* or child or teenager* or pediatric* or paediatric* or kid* or teen* or young person or young people or boy* or girl* or juvenile*).ti. or (child or adolescent).kw.
S3 (Mental Health)*	(attention deficit disorder* or attention deficit hyperactive disorder* or ADHD or Eating disorder* or eating problem* or Emerging personality disorder or emerging personality problem* or Externalising disorder* or externalising problem* or externalizing disorder* or externalizing problem* or Oppositional defiant disorder* or oppositional defiant problem* or Psychos* or psychotic disorder* or psychotic problem* or Anxiety or depression or depressive or obsessive compulsive disorder or OCD or phobia or phobic or mood disorder or anxiety disorder or panic disorder or agoraphobia or internalising problem* or internalising problem* or internalizing problem* or internalizing disorder*).ab. or (attention deficit disorder* or attention deficit hyperactive disorder* or ADHD or Eating disorder* or eating problem* or Emerging personality disorder or emerging personality problem* or Externalising disorder* or externalising problem* or externalizing disorder* or externalizing problem* or Oppositional defiant disorder* or oppositional defiant problem* or Psychos* or psychotic disorder* or psychotic problem* or Anxiety or depression or depressive or obsessive compulsive disorder or OCD or phobia or phobic or mood disorder or anxiety disorder or panic disorder or agoraphobia or internalising problem* or internalising problem* or internalizing problem* or internalizing disorder*).ti.
S4 (Needs)	(information need* or knowledge need* or need* or support or experience*or impact or wellbeing or concern* or want or perspective* or belief* or attitude* or prefer* or anxiety or anxious or depressed or depression or strain or stress or burden or parent satisfaction or family relationship or parent* self-efficacy).ab. or (information need* or knowledge need* or need* or support or experience*or impact or wellbeing or concern* or want or perspective* or belief* or attitude* or prefer* or anxiety or anxious or depressed or depression or strain or stress or burden or parent satisfaction or family relationship or parent* self-efficacy).ti.
S5	S1 adj8 s4
S6	S2 adj8 s5
S7	S5 and s6

Database: WoS

Data conducted the search on 14th November 2022

Search	Terms
1	TS=(parent OR parents OR parental OR mother OR father OR care*giver OR guardian* OR carer* OR paternal OR maternal)
2	TS=(children OR adolescent* OR adolescence OR youth* OR child OR teenager* OR pediatric* OR paediatric* OR kid* OR teen* OR 'young person' OR 'young people' OR boy* OR girl* OR juvenile*)

3	TS=('attention deficit disorder*' OR 'attention deficit hyperactive disorder*' OR adhd OR 'attention deficit disorder with hyperactivity' OR 'eating disorder*' OR 'eating problem*' OR 'feeding and eating disorders' OR 'emerging personality disorder*' OR 'emerging personality problem*' OR 'personality disorder' OR 'externalising disorder*' OR 'externalising problem*' OR 'externalizing disorder*' OR 'externalizing problem*' OR 'oppositional defiant disorder*' OR 'oppositional defiant problem*' OR 'attention deficit and disruptive behavior disorders' OR psychos* OR 'psychotic disorder*' OR 'psychotic problem*' OR anxiety OR depression OR depressive OR 'obsessive compulsive disorder' OR 'ocd' OR phobia OR phobic OR 'mood disorder' OR 'anxiety disorder' OR 'panic disorder' OR agoraphobia OR 'internalising problem*' OR 'internalising disorder*' OR 'internalizing problem*' OR 'internalizing disorder*)
4	TS=("Information need*" or "knowledge need*" or need* or support or experience*or impact or wellbeing or concern* or want or perspective* or belief* or attitude*or prefer* or anxiety or anxious or depressed or depression or strain or stress or burden or "parent satisfaction" or "family relationship" or "parent* self-efficacy")
5	TS=((parent OR parents OR parental OR mother OR father OR care*giver OR guardian* OR carer* OR paternal OR maternal) NEAR/8 ("Information need*" or "knowledge need*" or support or experience* OR impact* or wellbeing or concern* or want or perspective* or belief* or attitude* or prefer* or anxiety or anxious or depressed or depression or strain or stress or burden or "parent satisfaction" or "family relationship" or "parent* self-efficacy"))
6	TS=(("attention deficit disorder" or "attention deficit hyperactive disorder" or adhd or "attention deficit disorder with hyperactivity" or "eating disorder" or "eating problem" or "feeding and eating disorder" or "emerging personality disorder" or "emerging personality problem" or "personality disorder" or "externalising disorder" or "externalising problem" or "oppositional defiant disorder" or "oppositional defiant problem" or "attention deficit and disruptive behavior disorders" or psychos* or "psychotic" or anxiety or depression or depressive or anxious or "obsessive compulsive disorder" or OCD or phobia or phobic or "mood disorder" or "anxiety disorder" or "panic disorder" or agoraphobia or "internalising problem" or "internalising disorder" or "internalizing problem" or "internalizing disorder" or "externalizing problem" or "externalizing disorder") NEAR/8 (children or adolescent* or adolescence or youth* or child or teenager* or pediatric* or paediatric* or kid* or teen* or "young person" or "young people" or boy* or girl* or juvenile*))
7	#5 AND #6
8	#7 English
