# Supplementary material for: Needs of parents and carers of children and young people with mental health difficulties: protocol for a systematic review
Source: BMJ Open. 2023 Feb 16;13(2):e071341. doi: 10.1136/bmjopen-2022-071341 (PMC9936057; doi:10.1136/bmjopen-2022-071341)
Supplement: Author's manuscript [file bmjopen-2022-071341.draft_revisions.pdf]

BMJ Open is committed to open peer review. As part of this commitment we make the peer review history of every article we publish publicly available.

When an article is published we post the peer reviewers' comments and the authors' responses online. We also post the versions of the paper that were used during peer review. These are the versions that the peer review comments apply to.

The versions of the paper that follow are the versions that were submitted during the peer review process. They are not the versions of record or the final published versions. They should not be cited or distributed as the published version of this manuscript.

BMJ Open is an open access journal and the full, final, typeset and author-corrected version of record of the manuscript is available on our site with no access controls, subscription charges or pay-per-view fees (http://bmjopen.bmj.com).

If you have any questions on BMJ Open's open peer review process please email info.bmjopen@bmj.com

BMJ Open

# A systematic review protocol: Needs of parents` and carers` of children and young person with mental health difficulties

| | |
|---|---|
| Journal: | *BMJ Open* |
| Manuscript ID | bmjopen-2022-071341 |
| Article Type: | Protocol |
| Date Submitted by the Author: | 29-Dec-2022 |
| Complete List of Authors: | Martin, Faith; Cardiff University<br>Dahmash, Dania; Coventry University<br>Glover, Sarah; Parental Minds<br>Duncan, Charlie; British Association for Counselling and Psychotherapy<br>Turner, Andy; Coventry University<br>Halligan, Sarah; University of Bath, Department of Psychology |
| Keywords: | MENTAL HEALTH, Child & adolescent psychiatry < PSYCHIATRY, PUBLIC HEALTH |
| | |

SCHOLARONE™
Manuscripts

# A systematic review protocol: Needs of parents` and carers` of children and young person with mental health difficulties

**Authors**

Dr Faith Martin. School of Psychology, Cardiff University, 70 Park Pl, Cardiff CF10 3AS. Email martinf8@cardiff.ac.uk

Dr Dania Dahmash. Coventry University, Centre for Intelligent Healthcare, Coventry University, Richard Crossman Building, Jordan Well, Coventry CV1 5RW, UK. Email: ad9819@coventry.ac.uk

Mrs Sarah Glover. Parental Minds Community Interest Company, UK. Email: parentplus@parentalminds.org.uk

Charlie Duncan. Research Department, BACP House, 15 St John's Business Park, Lutterworth, LE17 4HB Email charlie.duncan@bacp.co.uk

Professor Andy Turner. Coventry University, Centre for Intelligent Healthcare, Coventry University, Richard Crossman Building, Jordan Well, Coventry CV1 5RW, UK. Email: hsx116@coventry.ac.uk

Professor Sarah Halligan. University of Bath, Department of Psychology, University of Bath, Claverton Down Campus, Bath BA2 7AY, UK. Email: slh54@bath.ac.uk

**Corresponding author:**

Dr Dania Dahmash. Coventry University, Centre for Intelligent Healthcare, Coventry University, Richard Crossman Building, Jordan Well, Coventry CV1 5RW, UK. Email ad9818@coventry.ac.uk



For peer review only - http://bmjopen.bmj.com/site/about/guidelines.xhtml

**Abstract:**

**Introduction:** Having a child or young person (CYP) with mental health problems can be highly distressing for parents/carers. The impact can include parental/carer depression, anxiety, lost productivity, and poor family relationships. Currently there is no synthesis of this evidence. which is needed to provide clarity around what support parents/carers may need, to meet the needs of family mental health. This review aims to identify the needs of the parents/carers of CYP who are receiving mental health services.

***Methods and analysis:*** A systematic review following the PRISMA-P guidelines will be conducted to identify potentially relevant studies that provide evidence concerning the needs and impact on parents/carers linked to their CYP having a mental health difficulty. CYP mental health conditions included are anxiety disorders, depression, psychoses, oppositional defiant and other externalising disorders, labels of emerging personality disorders, eating disorders, and attention deficit (hyperactive) disorders. The following databases will be searched: Medline; PsycInfo; CINAHL; AMED; EMBASE; Web of Science; Cochrane Library; WHO International Clinical Trials Registry Platform; Social Policy and Practice; Applied Social Sciences Index and Abstracts; and Open Grey. The quality of the included studies will be assessed using Joanna Briggs Institute Critical Appraisal Checklist for qualitative studies and the Newcastle Ottawa Scale for quantitative studies. Qualitative data will be analysed thematically and inductively.

**Ethical consideration and dissemination:** This review was approved by the ethical committee at Coventry university, UK. The findings from this systematic review will be disseminated across various key stakeholders and published in peer reviewed journals.

**Keywords**

Children; Young people; Mental health; Parental needs; Parents; Carers; Systematic review

For peer review only

**Article summary section:**

1- Demand from families for Child and Adolescent Mental Health Services (CAMHS) service is greater than ever before, with approximately one in six school-aged children having a mental health problem. Large numbers of parents/carers are currently supporting children with mental health difficulties, with limited professional input at times and high stress placed on them. This review to understand their needs is then timely.

2- This is the first review to understand the impact of having a child with a mental health difficulty on the parent/carers and represents a major step to better supporting parents/carers by covering knowledge and future research around parents/carers needs

3- We plan a thorough search strategy, including and synthesising qualitative and quantitative data to provide an in-depth picture of the current state of knowledge in this area.

4- The findings will be shared with various key stakeholder, to inform their research recommendations.

For peer review only



For peer review only - http://bmjopen.bmj.com/site/about/guidelines.xhtml

## INTRODUCTION

Almost 1,250,000 5- to 19-year-olds in the United Kingdom (UK) alone have a diagnosable mental illness and statutorily-funded UK Child and Adolescent Mental Health Services (CAMHS) have capacity to see just one third of these (1). Before COVID19, the prevalence of mental health disorders among 5–16-year-olds was 10%, which has increased to an estimated 16% post COVID-19 (2). This means greater demand on already stretched CAMHS, and more parents/carers trying to provide support.

There is a close association between children and young people's (CYP's) mental health (MH) and that of their parents/carers (3). Rates of diagnosed depression and other mental health problems are higher among parents/carers for CYP with MH problems compared to other parents: 51% compared to 23% (4-6). Having a child with a MH problem can be distressing for parents/carers, impacting their wellbeing, stress, and relationship with their CYP (7, 8). This leads to greater service use by parents/carers for their own psychological wellbeing and lost productivity through time off work. It is critical that we support parents/carers, in relation to their individual needs, the impact on the wider family, and for the indirect effect on the CYP.

Unfortunately, parents/carers are typically left needing information, support in helping their young people, and emotional support for themselves with the impact of seeing their young person in distress (8-10). The impact of CYP MH on parents/carers is acknowledged, for example the recent UK Care Quality Commission report suggests parents/carers be seen as partners in CYP MH care (11). There is then growing policy attention on supporting parents/carers, but a lack of consideration of the parents'/carers' own needs and wellbeing, as part of the family system. Improving CAMHS with an "integrated whole family approach" and greater support for parents/carers in supporting their CYP have been proposed (6, 12). To achieve this, an understanding of the impact of CYP MH problems on parents/carers is essential, to inform interventions. Currently a synthesised understanding is absent from treatment and clinical guidelines and there are no literature reviews that provide guidance on how to support these parents/carers. This proposal was designed to meet this critical need.

For peer review only

Hence, this literature review aims to synthesise current evidence in relation to parents'/carers' needs of young people who are receiving mental services, in order to then develop and improve interventions to support these parents/carers.

## METHOD AND ANALYSIS

The study has been approved by the ethical committee at Coventry University, UK (Reference number: P139611). This protocol follows the Preferred Reporting Items for Systematic Review and Meta-Analyses for Protocols (PRISMA-P) 2015 guidelines(13).

### Setting and participants

The age range of CYP is defined as 5 to 18 to reflect the upper limit of most commissioned CAMHS services in the UK. The lowest limit reflects the lower limit of the age of onset of most conditions (14) and the lower bound of UK surveillance data (15). Studies where the majority (over 50%) of the sample are parents/carers of CYP aged 5-18 will be included.

The term 'parents/carers' includes biological and step-parents, other relatives assuming a parenting role, non-biological and adoptive parents, foster-carers, and other adults in legal guardian roles. The CYP may have any mental health condition, including depression, anxiety disorders, psychoses, oppositional defiant and other externalising disorders, labels of emerging personality disorders, eating disorders, and attention deficit (hyperactive) disorders. This review does not cover issues in relation to primary prevention of mental health difficulties in childhood. The review does not cover parents/carers of children solely with special educational needs, including autism spectrum conditions or developmental language disorder. Studies with samples exclusively focused on parents/carers of CYP with these conditions will be excluded. Studies that focus on CYP with mental health difficulties and within that include less than 50% CYP with developmental/special educational needs would be included.

### Eligibility criteria

For peer review only



For peer review only - http://bmjopen.bmj.com/site/about/guidelines.xhtml

To be included in this review, studies must include participants who are parents/carers of CYP (aged 5-18), where the CYP has a mental health difficulty (as detailed above). The CYP must have received formal diagnosis and/or have accessed mental health services, be that via community services, primary care mental health services, secondary, inpatient, specialist services, or via health-care professionals in schools. Studies must provide primary quantitative or qualitative data relating to parents'/carers' needs or impact on parents/carers from CYPs MH difficulty. Outcomes or observations must relate to the impact of having a CYP with a mental health problem on the parents/carers and the needs they have in relation to that impact. Outcomes in quantitative studies must use validated measures only. Information and knowledge needs; parents'/carers' own mental health needs in relation to depression, anxiety, and stress; and parents'/carers' difficulties with poor parenting satisfaction, family relationship problems, and low parenting self-efficacy will be included. Only studies published in English will be included due to limited resources. No date restriction will be applied. A PRISMA flowchart will detail the complete study selection process.

**Study design**

Studies of any design, other than systematic reviews, are eligible for inclusion.

**Intervention and outcome**

This review focuses on understanding the impact on parents/carers of having a CYP with a mental health difficulty, and research that seeks to specify their needs. As such, studies may include interventions, however for this review we will only extract data where this describes need/impact. Quantitative studies must use validated measures, and report either comparison to a control group or use measures that have well established norms/clinical cut-off scores. This is to allow us to interpret the extent of the impact or need in our target population. Relevant outcomes are parental/carer stress, burnout, depression, anxiety, and stress; and parents'/carers' parenting satisfaction, family relationships, and parenting self-efficacy.

**Search strategy**

For peer review only



For peer review only - http://bmjopen.bmj.com/site/about/guidelines.xhtml

The following bibliographic database we will be searched: Medline; PsycInfo; CINAHL; AMED; EMBASE; Web of Science; Cochrane Library (including Cochrane Database of Systematic Reviews, Cochrane Central Register of Controlled Trials, Database of Abstracts of Reviews of Effects, Health Technology Assessment Database, and NHS Economic Evaluation Database); WHO International Clinical Trials Registry Platform (searches multiple databases including clinicaltrials.gov); Social Policy and Practice; Applied Social Sciences Index and Abstracts; and Open Grey.

The reference lists of relevant systematic reviews will be screened for additional studies not captured by the database searching. Cited reference searches and examination of reference lists will be conducted for related systematic reviews and any key papers identified.

The search terms will include four blocks of terms **(Table 1)**, all of which must be present, each relating to a key element of the review scope:

1. Parents or carers
2. Children and young people
3. Mental health problems
4. Needs or Impact

**Table 1:Search strategy to be applied in Medline database**

| **Search** | **Terms** |
|---|---|
| S1 (Parent) | TI ((parent or parents or parental or mother or father or care*giver or guardian* or carer* or paternal or maternal ) ) OR AB ( ( parent or parents or parental or mother or father or care*giver or guardian* or carer* or paternal or maternal ) ) OR MM ("Parents+") |
| S2 (Children) | TI ( (children or adolescent* or adolescence or youth* or child or teenager* or pediatric* or paediatric* or kid* or teen* or young person or young people or boy* or girl* or juvenile* ) ) OR AB ( ( children or adolescent* or adolescence or youth* or child or teenager* or pediatric* or paediatric* or kid* or teen* or young person or young people or boy* or girl* or juvenile* ) ) OR MH ("Child+") OR MM ("Adolescent") |
| S3 (Mental Health) | ( TI ( ( attention deficit disorder* or “attention deficit hyperactive disorder* “or “ADHD”) OR AB (attention deficit disorder* or “attention deficit hyperactive |

For peer review only



For peer review only - http://bmjopen.bmj.com/site/about/guidelines.xhtml

| | |
|---|---|
| | disorder* “or “ADHD”) OR MH (“Attention Deficit Disorder with Hyperactivity”) OR ( TI ( (Eating disorder* or eating problem*) ) OR AB ( (Eating disorder* or eating problem*) OR MH(“Feeding and Eating Disorders+”) OR ( TI ( (Emerging personality disorder* or emerging personality problem*) ) OR AB ( (Emerging personality disorder* or emerging personality problem*) ) OR MH (“Personality Disorder+”) ) OR ( TI ( (Externalising disorder* or externalising problem* or externalizing disorder* or externalizing problem*) OR AB (Externalising disorder* or externalising problem* or externalizing disorder* or externalizing problem*) OR TX (“Externalising disorder”) ) OR ( TI ( (Oppositional defiant disorder* or oppositional defiant problem*) ) OR AB ( (Oppositional defiant disorder* or oppositional defiant problem*) OR (MH“Attention Deficit and Disruptive Behavior Disorders+”) ) OR ( TI ( (Psychos* or psychotic disorder* or psychotic problem*) ) OR AB ( (Psychos* or psychotic disorder* or psychotic problem*) ) OR MH (“Psychotic Disorders+) ) OR ( TI ( ( Anxiety or depression or depressive or “obsessive compulsive disorder” or “OCD” or phobia or phobic or mood disorder or anxiety disorder or panic disorder or agoraphobia or internalising problem* or internalising problem* or internalizing problem* or internalizing disorder* ) ) OR AB ( ( Anxiety or depression or depressive or “obsessive compulsive disorder” or “OCD” or phobia or phobic or mood disorder or anxiety disorder or panic disorder or agoraphobia or internalising problem* or internalising problem* or internalizing problem* or internalizing disorder* ) OR (MH "Depressive Disorder") OR (MH "Depressive Disorder, Major") OR (MH "Depressive Disorder, Treatment-Resistant") OR (MH "Dysthymic Disorder") OR (MM "Anxiety Disorders+") ) |
| S4 (Needs or impact) | TI (“Information need*” or “knowledge need*” or need* or support or experience*or impact or wellbeing or concern* or want or perspective* or belief* or attitude*or prefer* or anxiety or anxious or depressed or depression or strain or stress or burden or “parent satisfaction” or “family relationship” or “parent* self-efficacy”) OR AB (“Information need*” or “knowledge need*” or need* or support or experience*or impact or wellbeing or concern* or want or perspective* or belief* or attitude*or prefer* or anxiety or anxious or depressed or depression or strain or stress or burden or “parent satisfaction” or “family relationship” or “parent* self-efficacy”)) |
| S5 | S1 N8 S4 |
| S6 | S2 N8 S3 |

## DATA COLLECTION AND ANALYSIS



For peer review only - http://bmjopen.bmj.com/site/about/guidelines.xhtml

**Selection process**

Rayyan software will be used to manage citations (16). It facilitates blind scoring for inclusion/exclusion. Inclusion/exclusion criteria will undergo final review by a Patient Public Involvement (PPI) group.

A two-step process will be used for screening. First, all titles and abstracts will be screened against the inclusion/exclusion criteria by two people independently. Second, full texts will be obtained for short-listed studies and formal inclusion/exclusion will be conducted by two reviewers independently. Any discrepancies will be resolved by a third reviewer if needed.

**Data extraction and quality appraisal**

Data will be extracted into Excel sheet following Cochrane guidelines and Joanna Briggs Institute (JBI) (17, 18). The data extraction sheet will be piloted using papers relevant to the research question and refined. Data extracted using the pilot form will then be re-checked using the final form.

All study characteristics (population, intervention, and comparator details) will be extracted, as well as study design, sample size, and sample characteristics (including parent/carer and their CYP characteristics). Quantitative data that captures needs or impact on parents/carers relating to their CYP's MH problems will be extracted. Quantitative studies with a single group of participants (parents/carers exposed to CYP self-harm) must use validated measures with established norms or clinical cut-offs, to allow interpretation of findings. Quantitative designs such as case-control, where a comparison group of parents/carers not exposed to CYP self-harm must use any validated measure, as levels of wellbeing etc in our target group can be interpreted in relation to the control group. Qualitative data describing parents'/carers' needs or the impact on them will be extracted in detail. Data extraction will include mean scores, standard deviations, percentage of participants in "clinical" cut-off range and effect sizes for case-control designs.

Quality appraisal will use JBI Critical Appraisal Checklist (17) (qualitative) and the Newcastle Ottawa Scale (19) (quantitative).



For peer review only - http://bmjopen.bmj.com/site/about/guidelines.xhtml

**Data synthesis**

Qualitative descriptions of impact or need will be synthesised thematically and inductively (17, 20). Two reviewers will do this independently, then discuss together, with decisions made with other reviewers and stakeholder input where needed (21). The impact on parents/carers and their needs will be visually mapped to capture any links between these. We will also group findings in relation to CYP diagnosis and examine differences relating to gender/identity of parent/carer.

Quantitative reports will be grouped by variable measures (e.g. depression, satisfaction with parenting, information needs) and summarised, with critical reflection on methodological quality. We anticipate inconsistency between studies in relation to measures used and comparison groups, rendering quantitative data synthesis unlikely.

A summary list of needs/impacts will be created based on these findings. The stakeholders, including our parent/carer working group will provide input on the themes, proposed links between them, and any needs/impacts/populations they perceive as under-researched.

The quantitative data will be integrated with qualitative findings by using the convergent integrated approach of mixed methods reviews proposed by JBI. This method is appropriate as the research questions can be answered by both quantitative and qualitative synthesis. Synthesis will be achieved by qualitizing quantitative findings into textural statements and pooling with qualitative data extracted directly from qualitative studies. Repeated, detailed examination of the assembled data will be undertaken to identify categories based on similarity in meaning. This will determine if results confirm/expand/refute one another. A category will integrate two or more qualitative data, qualitized data or a combination of both. Where possible, categories will be aggregated to produce overall finding(s). Where data cannot be categorised, findings will be reported narratively.

**Patient and Public involvement**

The scope of the review has been defined through discussion with Emerging Minds funded Special Interest Research Group (SIRG) on "Parents Support and Wellbeing", which includes psychologists, academics, parents/carers, and charity representatives. In addition, we consulted with parents/carers



For peer review only - http://bmjopen.bmj.com/site/about/guidelines.xhtml

who are members of parent support groups (for example, co-author SG's group "Parental Minds") and more widely across the UK's national network of parents' support groups (the "PLACE" network, hosted by our collaborators Charlie Waller Memorial Trust). The PPI group helped to design the study and specify the protocol, including the exclusion of autistic spectrum disorders and development difficulties from this review.

The PPI group will also be involved in the design of outputs and dissemination activities, including reviewing our interpretations for the formal reports and co-writing the outputs for a non-academic audience.

**DISSEMINATION:**

Dissemination will use multiple channels, including a project website, hosted by Coventry University. We will press release and widely disseminate through social media channels and YouTube, with support from the university's marketing department and our collaborating organisations' press offices.

We will disseminate findings via all our collaborators. Charlie Waller Trust's national network of parent support organisations and wider links to MH providers and trainers. Young Minds sit on several national MH advisory groups, will organise a government round table dissemination event, and publicise our findings across their widely accessed communication channels. The Emerging Minds Network also have a large audience of public, academics, and policy organisations (e.g. APPGs).

We will disseminate via the Future NHS Collaboration platform, which reaches clinicians, service leads, planners, commissioners and NHS England.

The Local Government Association have created the Mental Health Challenge (www.mentalhealthchallenge.org.uk) to lead and direct local council policy on mental health. We will use these channels to share our findings.

Parents/carers involved in the project will be sent the final outputs via email links/hard-copy. In addition, findings from this review will be published in a peer reviewed journal.



For peer review only - http://bmjopen.bmj.com/site/about/guidelines.xhtml

## ETHICAL CONSIDERATION

This study was approved by Coventry University ethical committee, reference number: P139611

## AUTHORS' CONTRIBUTIONS

FM created the idea and led the project, contributing to all stages. Study selection and data collection will be conducted by all authors. All authors will be involved in the synthesis and interpretation of the data. All authors and the parent/carer panel will be involved in the final drafting and dissemination of findings. All authors read and approved the final manuscript.

## AVAILABILITY OF DATA AND MATERIALS

Not applicable.

## FUNDING

This work was supported by the National Institute of Health Research (NIHR); grant number: NIHR20302.

## ACKNOWLEDGEMENTS

We would like to acknowledge and thank the members of the PPI group who have provided invaluable input to date.



For peer review only - http://bmjopen.bmj.com/site/about/guidelines.xhtml

## REFERENCES

1. Crenna-Jennings W, Hutchinson J. Access to child and adolescent mental health services in 2019. 2020.

2. Newlove-Delgado T, McManus S, Sadler K, Thandi S, Vizard T, Cartwright C, et al. Mental Health of Children and Young People group. 2021;8(5):353-4.

3. Campbell TC, Reupert A, Sutton K, Basu S, Davidson G, Middeldorp CM, et al. Prevalence of mental illness among parents of children receiving treatment within child and adolescent mental health services (CAMHS): a scoping review. 2021;30(7):997-1012.

4. Gerkensmeyer JE, Perkins SM, Day J, Austin JK, Scott EL, Wu JJJoC, et al. Maternal depressive symptoms when caring for a child with mental health problems. 2011;20(5):685-95.

5. Whitlock J, Lloyd-Richardson E, Fisseha F, Bates TJJocp. Parental secondary stress: The often hidden consequences of nonsuicidal self-injury in youth. 2018;74(1):178-96.

6. Education Df, Skills. Transforming Children and Young People's Mental Health Provision. 2017.



For peer review only - http://bmjopen.bmj.com/site/about/guidelines.xhtml

7. Arbuthnott AE, Lewis SPJC, psychiatry a, health m. Parents of youth who self-injure: a review of the literature and implications for mental health professionals. 2015;9(1):1-20.

8. Rodriguez-Meirinhos A, Antolin-Suarez L, Oliva AJAoPN. Support needs of families of adolescents with mental illness: A systematic mixed studies review. 2018;32(1):152-63.

9. Johnson SE, Lawrence D, Sawyer M, Zubrick SRJA, Psychiatry NZJo. Mental disorders in Australian 4-to 17-year olds: Parent-reported need for help. 2018;52(2):149-62.

10. Lawrence D, Johnson S, Hafekost J, Boterhoven de Haan K, Sawyer M, Ainley J, et al. The mental health of children and adolescents: Report on the second Australian child and adolescent survey of mental health and wellbeing. 2015.

11. Commission CQ. Are we listening? Review of children and young people's mental health services. Care Quality Commission London, UK; 2018.

12. Department of Health. Future in mind: Promoting, protecting and improving our children and young people's mental health and wellbeing. 2015.

13. Shamseer L, Moher D, Clarke M, Ghersi D, Liberati A, Petticrew M, et al. Preferred reporting items for systematic review and meta-analysis protocols (PRISMA-P) 2015: elaboration and explanation. 2015;349.

14. Kessler RC, Aguilar-Gaxiola S, Alonso J, Angermeyer MC, Anthony JC, Berglund PA, et al. Lifetime prevalence and age of onset distributions of mental disorders in the World Mental Health Survey Initiative. The WHO world mental health surveys: Global perspectives on the epidemiology of mental disorders: Cambridge University Press; 2008. p. 511-21.

15. Sadler K, Vizard T, Ford T, Marchesell F, Pearce N, Mandalia D, et al. Mental health of children and young people in England, 2017. 2018.

16. Ouzzani M, Hammady H, Fedorowicz Z, Elmagarmid AJSr. Rayyan—a web and mobile app for systematic reviews. 2016;5(1):1-10.

17. Moola S, Munn Z, Tufanaru C, Aromataris E, Sears K, Sfetcu R, et al. Chapter 7: Systematic reviews of etiology and risk. 2017;5.

18. Higgins J, Thomas J, Chandler J, Cumpston M, Li T, Page M, et al. Cochrane Handbook for Systematic Reviews of Interventions Version 6.2.



For peer review only - http://bmjopen.bmj.com/site/about/guidelines.xhtml

19. Wells GA, Shea B, O'Connell D, Peterson J, Welch V, Losos M, et al. The Newcastle-Ottawa Scale (NOS) for assessing the quality of nonrandomised studies in meta-analyses. Oxford; 2000.

20. Popay J, Roberts H, Sowden A, Petticrew M, Arai L, Rodgers M, et al. Guidance on the conduct of narrative synthesis in systematic reviews. 2006;1(1):b92.

21. Thomas J, Harden A. Methods for the thematic synthesis of qualitative research in systematic reviews. 2008;8(1):1-10.

For peer review only



For peer review only - http://bmjopen.bmj.com/site/about/guidelines.xhtml

# PRISMA-P 2015 Checklist

**This checklist has been adapted for use with systematic review protocol submissions to BioMed Central journals from Table 3 in Moher D et al:** Preferred reporting items for systematic review and meta-analysis protocols (PRISMA-P) 2015 statement. *Systematic Reviews* 2015 **4**:1

An Editorial from the Editors-in-Chief of *Systematic Reviews* details why this checklist was adapted - **Moher D, Stewart L & Shekelle P:** Implementing PRISMA-P: recommendations for prospective authors. *Systematic Reviews* 2016 **5**:15

| Section/topic | # | Checklist item | Information reported | | Line number(s) |
|---|---|---|---|---|---|
| | | | Yes | No | |
| **ADMINISTRATIVE INFORMATION** | | | | | |
| **Title** | | | | | |
| Identification | 1a | Identify the report as a protocol of a systematic review | ✓ | | 1 |
| Update | 1b | If the protocol is for an update of a previous systematic review, identify as such | | ✓ | Not applicable |
| **Registration** | 2 | If registered, provide the name of the registry (e.g., PROSPERO) and registration number in the Abstract | | ✓ | Not applicable, NIHRA Funded |
| **Authors** | | | | | |
| Contact | 3a | Provide name, institutional affiliation, and e-mail address of all protocol authors; provide physical mailing address of corresponding author | ✓ | | 4-15 |
| Contributions | 3b | Describe contributions of protocol authors and identify the guarantor of the review | ✓ | | 233-2347 |
| **Amendments** | 4 | If the protocol represents an amendment of a previously completed or published protocol, identify as such and list changes; otherwise, state plan for documenting important protocol amendments | | ✓ | Not applicable |
| **Support** | | | | | |
| Sources | 5a | Indicate sources of financial or other support for the review | ✓ | | 241-243 |
| Sponsor | 5b | Provide name for the review funder and/or sponsor | ✓ | | 241-243 |
| Role of sponsor/funder | 5c | Describe roles of funder(s), sponsor(s), and/or institution(s), if any, in developing the protocol | | ✓ | Not applicable |
| **INTRODUCTION** | | | | | |
| **Rationale** | 6 | Describe the rationale for the review in the context of what is already known | ✓ | | 73-100 |
| **Objectives** | 7 | Provide an explicit statement of the question(s) the review will address with reference to participants, interventions, comparators, and outcomes (PICO) | ✓ | | 98-100 |
| **METHODS** | | | | | |

BioMed Central
The Open Access Publisher

For peer review only - http://bmjopen.bmj.com/site/about/guidelines.xhtml

| Section/topic | # | Checklist item | Information reported Yes | No | Line number(s) |
|---|---|---|---|---|---|
| **Eligibility criteria** | 8 | Specify the study characteristics (e.g., PICO, study design, setting, time frame) and report characteristics (e.g., years considered, language, publication status) to be used as criteria for eligibility for the review | ✓ | | 121-134 |
| **Information sources** | 9 | Describe all intended information sources (e.g., electronic databases, contact with study authors, trial registers, or other grey literature sources) with planned dates of coverage | ✓ | | 146-155 |
| **Search strategy** | 10 | Present draft of search strategy to be used for at least one electronic database, including planned limits, such that it could be repeated | ✓ | | 146-163 |
| ***STUDY RECORDS*** | | | | | |
| Data management | 11a | Describe the mechanism(s) that will be used to manage records and data throughout the review | ✓ | | 166-172 |
| Selection process | 11b | State the process that will be used for selecting studies (e.g., two independent reviewers) through each phase of the review (i.e., screening, eligibility, and inclusion in meta-analysis) | ✓ | | 165-172 |
| Data collection process | 11c | Describe planned method of extracting data from reports (e.g., piloting forms, done independently, in duplicate), any processes for obtaining and confirming data from investigators | ✓ | | 173-189 |
| **Data items** | 12 | List and define all variables for which data will be sought (e.g., PICO items, funding sources), any pre-planned data assumptions and simplifications | ✓ | | 156-161 |
| **Outcomes and prioritization** | 13 | List and define all outcomes for which data will be sought, including prioritization of main and additional outcomes, with rationale | ✓ | | 174-187 |
| **Risk of bias in individual studies** | 14 | Describe anticipated methods for assessing risk of bias of individual studies, including whether this will be done at the outcome or study level, or both; state how this information will be used in data synthesis | ✓ | | 188-189 |
| ***DATA*** | | | | | |
| **Synthesis** | 15a | Describe criteria under which study data will be quantitatively synthesized | ✓ | | 190-211 |
| | 15b | If data are appropriate for quantitative synthesis, describe planned summary measures, methods of handling data, and methods of combining data from studies, including any planned exploration of consistency (e.g., $I^2$, Kendall's tau) | ✓ | | 190-211 |
| | 15c | Describe any proposed additional analyses (e.g., sensitivity or subgroup analyses, meta-regression) | ✓ | | 190-211 |
| | 15d | If quantitative synthesis is not appropriate, describe the type of summary planned | ✓ | | 211 |
| **Meta-bias(es)** | 16 | Specify any planned assessment of meta-bias(es) (e.g., publication bias across studies, selective reporting within studies) | ✓ | | 188-189 |
| **Confidence in cumulative evidence** | 17 | Describe how the strength of the body of evidence will be assessed (e.g., GRADE) | ✓ | | 188-189 |

BioMed Central
The Open Access Publisher

BMJ Open

# Needs of parents and carers of children and young people with mental health difficulties: protocol for a systematic review

| | |
|---|---|
| Journal: | *BMJ Open* |
| Manuscript ID | bmjopen-2022-071341.R1 |
| Article Type: | Protocol |
| Date Submitted by the Author: | 08-Feb-2023 |
| Complete List of Authors: | Martin, Faith; Cardiff University<br>Dahmash, Dania; Coventry University<br>Glover, Sarah; Parental Minds<br>Duncan, Charlie; British Association for Counselling and Psychotherapy<br>Turner, Andy; Coventry University<br>Halligan, Sarah; University of Bath, Department of Psychology |
| <b>Primary Subject Heading</b>: | Public health |
| Secondary Subject Heading: | Mental health, Public health, Paediatrics |
| Keywords: | MENTAL HEALTH, Child & adolescent psychiatry < PSYCHIATRY, PUBLIC HEALTH |
| | |

SCHOLARONE™
Manuscripts

# Needs of parents and carers of children and young people with mental health difficulties: protocol for a systematic review

Dr Faith Martin, School of Psychology, Cardiff University, 70 Park Pl, Cardiff, UK

martinf8@cardiff.ac.uk

Dr Dania Dahmash, Centre for Intelligent Healthcare, Coventry University, Richard Crossman Building, Jordan Well, Coventry, UK

ad9819@coventry.ac.uk

Mrs Sarah Glover, Parental Minds Community Interest Company, UK

parentplus@parentalminds.org.uk

Charlie Duncan, Research Department, BACP House, 15 St John's Business Park, Lutterworth, UK

charlie.duncan@bacp.co.uk

Professor Andy Turner, Centre for Intelligent Healthcare, Coventry University, Richard Crossman Building, Jordan Well, Coventry, UK

hsx116@coventry.ac.uk

Professor Sarah Halligan, Department of Psychology, University of Bath, Claverton Down Campus, Bath, UK

slh54@bath.ac.uk

**Correspondence to:**

Dr Dania Dahmash

Centre for Intelligent Healthcare, Coventry University, Richard Crossman Building, Jordan Well, Coventry CV1 5RW, UK.

ad9818@coventry.ac.uk

## Abstract

For peer review only

**Introduction:** Having a child or young person (CYP) with mental health problems can be highly distressing for parents/carers. The impact can include parental/carer depression, anxiety, lost productivity, and poor family relationships. Currently, there is no synthesis of this evidence, which is needed to provide clarity around what support parents/carers may need, to meet the needs of family mental health. This review aims to identify the needs of the parents/carers of CYP who are receiving mental health services.

**Methods and analysis:** A systematic review will be conducted to identify potentially relevant studies that provide evidence concerning the needs and impact on parents/carers linked to their CYP having mental health difficulties. CYP mental health conditions included are anxiety disorders, depression, psychoses, oppositional defiant and other externalising disorders, labels of emerging personality disorders, eating disorders, and attention deficit (hyperactive) disorders. The following databases were searched on November 2022 with no date restriction applied: Medline; PsycInfo; CINAHL; AMED; EMBASE; Web of Science; Cochrane Library; WHO International Clinical Trials Registry Platform; Social Policy and Practice; Applied Social Sciences Index and Abstracts; and Open Grey. Only studies reported in English will be included. The quality of the included studies will be assessed using Joanna Briggs Institute Critical Appraisal Checklist for qualitative studies and the Newcastle Ottawa Scale for quantitative studies. Qualitative data will be analysed thematically and inductively.

**Ethics and dissemination:** This review was approved by the ethical committee at Coventry University, UK, reference number P139611. The findings from this systematic review will be disseminated across various key stakeholders and published in peer-reviewed journals.

**Keywords**

Children; Young people; Mental health; Parental needs; Parents; Carers; Systematic review

**Strengths and limitations of this study**

For peer review only



For peer review only - http://bmjopen.bmj.com/site/about/guidelines.xhtml

- This systematic review protocol follows the Preferred Reporting Items for Systematic Reviews and Meta-Analysis Protocols guidelines.
- Qualitative and quantitative studies will be included to provide a comprehensive review of the impact on and needs of parents/carers of young people with mental health difficulties.
- The review will be limited to studies published in English, raising a potential language bias.

For peer review only



For peer review only - http://bmjopen.bmj.com/site/about/guidelines.xhtml

## INTRODUCTION

Almost 1,250,000 5- to 19-year-olds in the United Kingdom (UK) alone have a diagnosable mental illness and statutorily-funded UK Child and Adolescent Mental Health Services (CAMHS) have capacity to see just one third of these (1). Before COVID19, the prevalence of mental health disorders among 5–16-year-olds was 10%, which has increased to an estimated 16% post COVID-19 (2). This means greater demand on already stretched CAMHS, and more parents/carers trying to provide support.

There is a close association between children and young people's (CYP's) mental health (MH) and that of their parents/carers (3). Rates of diagnosed depression and other mental health problems are higher among parents/carers for CYP with MH problems compared to other parents: 51% compared to 23% (4-6). Having a child with a MH problem can be distressing for parents/carers, impacting their wellbeing, stress, and relationship with their CYP (7, 8). This leads to greater service use by parents/carers for their own psychological wellbeing and lost productivity through time off work. It is critical that we support parents/carers, in relation to their individual needs, the impact on the wider family, and for the indirect effect on the CYP.

Unfortunately, parents/carers are typically left needing information, support in helping their young people, and emotional support for themselves with the impact of seeing their young person in distress (8-10). The impact of CYP MH on parents/carers is acknowledged, for example the recent UK Care Quality Commission report suggests parents/carers be seen as partners in CYP MH care (11). There is then growing policy attention on supporting parents/carers, but a lack of consideration of the parents'/carers' own needs and wellbeing, as part of the family system. Improving CAMHS with an "integrated whole family approach" and greater support for parents/carers in supporting their CYP have been proposed (6, 12). To achieve this, an understanding of the impact of CYP MH problems on parents/carers is essential, to inform interventions. Currently a synthesised understanding is absent from treatment and clinical guidelines and there are no literature reviews that provide guidance on how to support these parents/carers. This proposal was designed to meet this critical need.



For peer review only - http://bmjopen.bmj.com/site/about/guidelines.xhtml

Hence, this systematic review aims to synthesise current evidence in relation to parents'/carers' needs of young people who are receiving mental services, in order to then develop and improve interventions to support these parents/carers.

## METHOD AND ANALYSIS

This systematic review has been approved by the ethical committee at Coventry University, UK (reference number P139611). This protocol follows the Preferred Reporting Items for Systematic Review and Meta-Analyses for Protocols (PRISMA-P) 2015 guidelines(13).

The age range of CYP is defined as 5 to 18 to reflect the upper limit of most commissioned CAMHS services in the UK. The lowest limit reflects the lower limit of the age of onset of most conditions (14) and the lower bound of UK surveillance data (15). Studies where the majority (over 50%) of the sample are parents/carers of CYP aged 5-18 will be included.

The term 'parents/carers' includes biological and step-parents, other relatives assuming a parenting role, non-biological and adoptive parents, foster carers, and other adults in legal guardian roles. The CYP may have any mental health condition, including depression, anxiety disorders, psychoses, oppositional defiant and other externalising disorders, labels of emerging personality disorders, eating disorders, and attention deficit (hyperactive) disorders. This review does not cover issues in relation to the primary prevention of mental health difficulties in childhood. The review does not cover parents/carers of children solely with special educational needs, including autism spectrum conditions or developmental language disorders. Studies with samples exclusively focused on parents/carers of CYP with these conditions will be excluded. Studies that focus on CYP with mental health difficulties and within that include less than 50% CYP with developmental/special educational needs would be included.

### Eligibility criteria

To be included in this review, studies must include participants who are parents/carers of CYP (aged 5-18), where the CYP has a mental health difficulty (as detailed above). The CYP must have received a



For peer review only - http://bmjopen.bmj.com/site/about/guidelines.xhtml

formal diagnosis and/or have accessed mental health services, be that via community services, primary care mental health services, secondary, inpatient, specialist services, or via health-care professionals in schools. Studies must provide primary quantitative or qualitative data relating to parents'/carers' needs or impact on parents/carers from CYPs MH difficulty. Outcomes or observations must relate to the impact of having a CYP with a mental health problem on the parents/carers and the needs they have in relation to that impact. Outcomes in quantitative studies must use validated measures only. Information and knowledge needs; parents'/carers' own mental health needs in relation to depression, anxiety, and stress; and parents'/carers' difficulties with poor parenting satisfaction, family relationship problems, and low parenting self-efficacy will be included. Only studies published in English will be included due to limited resources. No date restriction will be applied. A PRISMA flowchart will detail the complete study selection process.

**Study designs**

Studies of any design, other than systematic reviews, are eligible for inclusion.

**Interventions and outcomes**

This review focuses on understanding the impact on parents/carers of having a CYP with a mental health difficulty, and research that seeks to specify their needs. As such, studies may include interventions, however, for this review, we will only extract data where this describes need/impact. Quantitative studies must use validated measures, and report either comparison to a control group or use measures that have well established norms/clinical cut-off scores. This is to allow us to interpret the extent of the impact or need in our target population. Relevant outcomes are parental/carer stress, burnout, depression, anxiety, and stress; and parents'/carers' parenting satisfaction, family relationships, and parenting self-efficacy.

**Search strategy**

The following bibliographic databases were searched from inception until November 2022: Medline; PsycInfo; CINAHL; AMED; EMBASE; Web of Science; Cochrane Library (including Cochrane Database of Systematic Reviews, Cochrane Central Register of Controlled Trials, Database of Abstracts



For peer review only - http://bmjopen.bmj.com/site/about/guidelines.xhtml

of Reviews of Effects, Health Technology Assessment Database, and NHS Economic Evaluation Database); WHO International Clinical Trials Registry Platform (searches multiple databases including clinicaltrials.gov); Social Policy and Practice; Applied Social Sciences Index and Abstracts; and Open Grey. The search strategy for Medline is shown in **Table 1**, and the full search strategies for all databases are shown in **Supplementary File 1**.

The reference lists of relevant systematic reviews will be screened for additional studies not captured by the database search. Cited reference searches and examination of reference lists will be conducted for related systematic reviews and any key papers identified.

The search terms will include four blocks of terms (Table 1), all of which must be present, each relating to a key element of the review scope:

1. Parents or carers
2. Children and young people
3. Mental health problems
4. Needs or Impact

**Table 1. Search strategy for Medline**

| Search | Terms |
|---|---|
| S1 (Parent) | TI ((parent or parents or parental or mother or father or care*giver or guardian* or carer* or paternal or maternal ) ) OR AB ( ( parent or parents or parental or mother or father or care*giver or guardian* or carer* or paternal or maternal ) ) OR MM ("Parents+") |
| S2 (Children) | TI ( (children or adolescent* or adolescence or youth* or child or teenager* or pediatric* or paediatric* or kid* or teen* or young person or young people or boy* or girl* or juvenile* ) ) OR AB ( ( children or adolescent* or adolescence or youth* or child or teenager* or pediatric* or paediatric* or kid* or teen* or young person or young people or boy* or girl* or juvenile* ) ) OR MH ("Child+") OR MM ("Adolescent") |
| S3 (Mental Health) | ( TI ( ( attention deficit disorder* or “attention deficit hyperactive disorder* “or “ADHD”) OR AB (attention deficit disorder* or “attention deficit hyperactive disorder* “or “ADHD”) OR MH (“Attention Deficit Disorder with Hyperactivity”) |

For peer review only



For peer review only - http://bmjopen.bmj.com/site/about/guidelines.xhtml

| | |
|---|---|
| | OR ( TI ( (Eating disorder* or eating problem*) ) OR AB ( (Eating disorder* or eating problem*) OR MH("Feeding and Eating Disorders+") OR ( TI ( (Emerging personality disorder* or emerging personality problem*) ) OR AB ( (Emerging personality disorder* or emerging personality problem*) ) OR MH ("Personality Disorder+") ) OR ( TI ( (Externalising disorder* or externalising problem* or externalizing disorder* or externalizing problem*) OR AB (Externalising disorder* or externalising problem* or externalizing disorder* or externalizing problem*) OR TX ("Externalising disorder") ) OR ( TI ( (Oppositional defiant disorder* or oppositional defiant problem*) ) OR AB ( (Oppositional defiant disorder* or oppositional defiant problem*) OR (MH"Attention Deficit and Disruptive Behavior Disorders+") ) OR ( TI ( (Psychos* or psychotic disorder* or psychotic problem*) ) OR AB ( (Psychos* or psychotic disorder* or psychotic problem*) ) OR MH ("Psychotic Disorders+) ) OR ( TI ( ( Anxiety or depression or depressive or "obsessive compulsive disorder" or "OCD" or phobia or phobic or mood disorder or anxiety disorder or panic disorder or agoraphobia or internalising problem* or internalising problem* or internalizing problem* or internalizing disorder* ) ) OR AB ( ( Anxiety or depression or depressive or "obsessive compulsive disorder" or "OCD" or phobia or phobic or mood disorder or anxiety disorder or panic disorder or agoraphobia or internalising problem* or internalising problem* or internalizing problem* or internalizing disorder* ) OR (MH "Depressive Disorder") OR (MH "Depressive Disorder, Major") OR (MH "Depressive Disorder, Treatment-Resistant") OR (MH "Dysthymic Disorder") OR (MM "Anxiety Disorders+") ) |
| S4 (Needs or impact) | TI ("Information need*" or "knowledge need*" or need* or support or experience*or impact or wellbeing or concern* or want or perspective* or belief* or attitude*or prefer* or anxiety or anxious or depressed or depression or strain or stress or burden or "parent satisfaction" or "family relationship" or "parent* self-efficacy") OR AB ("Information need*" or "knowledge need*" or need* or support or experience*or impact or wellbeing or concern* or want or perspective* or belief* or attitude*or prefer* or anxiety or anxious or depressed or depression or strain or stress or burden or "parent satisfaction" or "family relationship" or "parent* self-efficacy")) |
| S5 | S1 N8 S4 |
| S6 | S2 N8 S3 |

164

165 **Selection process**



For peer review only - http://bmjopen.bmj.com/site/about/guidelines.xhtml

Rayyan software will be used to manage citations (16). It facilitates blind scoring for inclusion/exclusion. Inclusion/exclusion criteria will undergo a final review by a Patient Public Involvement (PPI) group.

A two-step process will be used for screening. First, all titles and abstracts will be screened against the inclusion/exclusion criteria by two people independently. Second, full texts will be obtained for short-listed studies and formal inclusion/exclusion will be conducted by two reviewers independently. Any discrepancies will be resolved by a third reviewer if needed.

**Data extraction and quality appraisal**

Data will be extracted into an Excel sheet following Cochrane guidelines and Joanna Briggs Institute (JBI) (17, 18). The data extraction sheet will be piloted using papers relevant to the research question and refined. Data extracted using the pilot form will then be re-checked using the final form.

All study characteristics (population, intervention, and comparator details) will be extracted, as well as study design, sample size, and sample characteristics (including parent/carer and their CYP characteristics). Quantitative data that captures the needs or impact of parents/carers relating to their CYP's MH problems will be extracted. Quantitative studies with a single group of participants (parents/carers exposed to CYP self-harm) must use validated measures with established norms or clinical cut-offs, to allow interpretation of findings. Quantitative designs such as case-control, where a comparison group of parents/carers not exposed to CYP self-harm must use any validated measure, as levels of wellbeing etc in our target group can be interpreted in relation to the control group. Qualitative data describing parents'/carers' needs or their impact on them will be extracted in detail. Data extraction will include mean scores, standard deviations, percentage of participants in the "clinical" cut-off range and effect sizes for case-control designs.

For the quality appraisal, the following tools will used: JBI Critical Appraisal Checklist (17) for the qualitative studies and the Newcastle Ottawa Scale (19) for quantitative studies.

**Data synthesis**



For peer review only - http://bmjopen.bmj.com/site/about/guidelines.xhtml

Qualitative descriptions of impact or need will be synthesised thematically and inductively (17, 20). Two reviewers will do this independently, then discuss together, with decisions made with other reviewers and stakeholder input where needed (21). The impact on parents/carers and their needs will be visually mapped to capture any links between these. We will also group findings in relation to CYP diagnosis and examine differences relating to the gender/identity of parent/carer.

Quantitative reports will be grouped by variable measures (e.g. depression, satisfaction with parenting, information needs) and summarised, with critical reflection on methodological quality. We anticipate inconsistency between studies in relation to measures used and comparison groups, rendering quantitative data synthesis unlikely.

A summary list of needs/impacts will be created based on these findings. The stakeholders, including our parent/carer working group, will provide input on the themes, proposed links between them, and any needs/impacts/populations they perceive as under-researched.

The quantitative data will be integrated with qualitative findings by using the convergent integrated approach of mixed methods reviews proposed by JBI. This method is appropriate as the research questions can be answered by both quantitative and qualitative synthesis. The synthesis will be achieved by qualitizing quantitative findings into textural statements and pooling them with qualitative data extracted directly from qualitative studies. Repeated, a detailed examination of the assembled data will be undertaken to identify categories based on similarity in meaning. This will determine if the results confirm/expand/refute one another. A category will integrate two or more qualitative data, qualitized data or a combination of both. Where possible, categories will be aggregated to produce overall finding(s). Where data cannot be categorised, findings will be reported narratively.

**Patient and public involvement**

The scope of the review has been defined through discussion with Emerging Minds funded Special Interest Research Group (SIRG) on "Parents Support and Wellbeing", which includes psychologists, academics, parents/carers, and charity representatives. In addition, we consulted with parents/carers who are members of parent support groups (for example, co-author SG's group "Parental Minds") and



For peer review only - http://bmjopen.bmj.com/site/about/guidelines.xhtml

more widely across the UK's national network of parents' support groups (the "PLACE" network, hosted by our collaborators Charlie Waller Memorial Trust). The PPI group helped to design the study and specify the protocol, including the exclusion of autistic spectrum disorders and development difficulties from this review.

The PPI group will also be involved in the design of outputs and dissemination activities, including reviewing our interpretations for the formal reports and co-writing the outputs for a non-academic audience.

## ETHICS AND DISSEMINATION

This study was approved by Coventry University ethical committee, reference number P139611.

Dissemination will use multiple channels, including a project website, hosted by Coventry University. We will press release and widely disseminated through social media channels and YouTube, with support from the university's marketing department and our collaborating organisations' press offices.

We will disseminate findings via all our collaborators. Charlie Waller Trust's national network of parent support organisations and wider links to MH providers and trainers. Young Minds sit on several national MH advisory groups, will organise a government round table dissemination event, and publicise our findings across their widely accessed communication channels. The Emerging Minds Network also have a large audience of public, academics, and policy organisations (e.g. APPGs).

We will disseminate via the Future NHS Collaboration platform, which reaches clinicians, service leads, planners, commissioners, and NHS England.

The Local Government Association have created the Mental Health Challenge (www.mentalhealthchallenge.org.uk) to lead and direct local council policy on mental health. We will use these channels to share our findings.

Parents/carers involved in the project will be sent the final outputs via email links/hard-copy. In addition, findings from this review will be published in a peer reviewed journal.



For peer review only - http://bmjopen.bmj.com/site/about/guidelines.xhtml

*** ***

## COMPETING INTERESTS

All authors have completed the ICMJE uniform disclosure form. SG is an author and the director of Parental Minds, which provide services to parent of children with mental health. All other authors declare no competing interests.

## CONTRIBUTORS

FM created the idea and led the project, conduced the literature review and contributing to all stages. FM DD prepared the manuscript draft. SG, CD, AT and SH contributed in the writing and the reviewing of and the manuscript. Study selection and data collection will be conducted by FM and DD. FM, DD, SG, CD, AT will be involved in the synthesis and interpretation of the data. FM, DD, SG, CD, AT, and SH along with the parent/carer panel will be involved in the final drafting and dissemination of findings. FM, DD, SG, CD, AT, and SH read and approved the final manuscript.

## FUNDING

This work was supported by the UK National Institute for Health and Care Research (NIHR), grant number NIHR20302.

## ACKNOWLEDGEMENTS

We would like to acknowledge and thank the members of the PPI group who have provided invaluable input to date.

## REFERENCES



For peer review only - http://bmjopen.bmj.com/site/about/guidelines.xhtml

1. Crenna-Jennings W, Hutchinson J. Access to child and adolescent mental health services in 2019. 2020.

2. Newlove-Delgado T, McManus S, Sadler K, Thandi S, Vizard T, Cartwright C, et al. Mental Health of Children and Young People group. 2021;8(5):353-4.

3. Campbell TC, Reupert A, Sutton K, Basu S, Davidson G, Middeldorp CM, et al. Prevalence of mental illness among parents of children receiving treatment within child and adolescent mental health services (CAMHS): a scoping review. 2021;30(7):997-1012.

4. Gerkensmeyer JE, Perkins SM, Day J, Austin JK, Scott EL, Wu JJJoC, et al. Maternal depressive symptoms when caring for a child with mental health problems. 2011;20(5):685-95.

5. Whitlock J, Lloyd-Richardson E, Fisseha F, Bates TJJocp. Parental secondary stress: The often hidden consequences of nonsuicidal self-injury in youth. 2018;74(1):178-96.

6. Education Df, Skills. Transforming Children and Young People's Mental Health Provision. 2017.

7. Arbuthnott AE, Lewis SPJC, psychiatry a, health m. Parents of youth who self-injure: a review of the literature and implications for mental health professionals. 2015;9(1):1-20.

8. Rodriguez-Meirinhos A, Antolin-Suarez L, Oliva AJAoPN. Support needs of families of adolescents with mental illness: A systematic mixed studies review. 2018;32(1):152-63.

9. Johnson SE, Lawrence D, Sawyer M, Zubrick SRJA, Psychiatry NZJo. Mental disorders in Australian 4-to 17-year olds: Parent-reported need for help. 2018;52(2):149-62.

10. Lawrence D, Johnson S, Hafekost J, Boterhoven de Haan K, Sawyer M, Ainley J, et al. The mental health of children and adolescents: Report on the second Australian child and adolescent survey of mental health and wellbeing. 2015.

11. Commission CQ. Are we listening? Review of children and young people's mental health services. Care Quality Commission London, UK; 2018.

12. Department of Health. Future in mind: Promoting, protecting and improving our children and young people's mental health and wellbeing. 2015.

For peer review only



For peer review only - http://bmjopen.bmj.com/site/about/guidelines.xhtml

13. Shamseer L, Moher D, Clarke M, Ghersi D, Liberati A, Petticrew M, et al. Preferred reporting items for systematic review and meta-analysis protocols (PRISMA-P) 2015: elaboration and explanation. 2015;349.

14. Kessler RC, Aguilar-Gaxiola S, Alonso J, Angermeyer MC, Anthony JC, Berglund PA, et al. Lifetime prevalence and age of onset distributions of mental disorders in the World Mental Health Survey Initiative. The WHO world mental health surveys: Global perspectives on the epidemiology of mental disorders: Cambridge University Press; 2008. p. 511-21.

15. Sadler K, Vizard T, Ford T, Marchesell F, Pearce N, Mandalia D, et al. Mental health of children and young people in England, 2017. 2018.

16. Ouzzani M, Hammady H, Fedorowicz Z, Elmagarmid AJSr. Rayyan—a web and mobile app for systematic reviews. 2016;5(1):1-10.

17. Moola S, Munn Z, Tufanaru C, Aromataris E, Sears K, Sfetcu R, et al. Chapter 7: Systematic reviews of etiology and risk. 2017;5.

18. Higgins J, Thomas J, Chandler J, Cumpston M, Li T, Page M, et al. Cochrane Handbook for Systematic Reviews of Interventions Version 6.2.

19. Wells GA, Shea B, O'Connell D, Peterson J, Welch V, Losos M, et al. The Newcastle-Ottawa Scale (NOS) for assessing the quality of nonrandomised studies in meta-analyses. Oxford; 2000.

20. Popay J, Roberts H, Sowden A, Petticrew M, Arai L, Rodgers M, et al. Guidance on the conduct of narrative synthesis in systematic reviews. 2006;1(1):b92.

21. Thomas J, Harden A. Methods for the thematic synthesis of qualitative research in systematic reviews. 2008;8(1):1-10.

For peer review only



For peer review only - http://bmjopen.bmj.com/site/about/guidelines.xhtml

**Search conducted 8th November 2022 or thereabouts**

**Database:** Medline

**Date:** 8th November 2022

| Search | Terms |
|---|---|
| S1 (Parent) | TI ((parent or parents or parental or mother or father or care*giver or guardian* or carer* or paternal or maternal ) ) OR AB ( ( parent or parents or parental or mother or father or care*giver or guardian* or carer* or paternal or maternal ) ) OR MM ("Parents+") |
| S2 (Children) | TI ( (children or adolescent* or adolescence or youth* or child or teenager* or pediatric* or paediatric* or kid* or teen* or young person or young people or boy* or girl* or juvenile* ) ) OR AB ( ( children or adolescent* or adolescence or youth* or child or teenager* or pediatric* or paediatric* or kid* or teen* or young person or young people or boy* or girl* or juvenile* ) ) OR MH ("Child+") OR MM ("Adolescent") |
| S3 (Mental Health) | ( TI ( ( attention deficit disorder* or "attention deficit hyperactive disorder* "or "ADHD") OR AB (attention deficit disorder* or "attention deficit hyperactive disorder* "or "ADHD") OR MH ("Attention Deficit Disorder with Hyperactivity") OR ( TI ( (Eating disorder* or eating problem*) ) OR AB ( (Eating disorder* or eating problem*) OR MH("Feeding and Eating Disorders+") OR ( TI ( (Emerging personality disorder* or emerging personality problem*) ) OR AB ( (Emerging personality disorder* or emerging personality problem*) ) OR MH ("Personality Disorder+") ) OR ( TI ( (Externalising disorder* or externalising problem* or externalizing disorder* or externalizing problem*) OR AB (Externalising disorder* or externalising problem* or externalizing disorder* or externalizing problem*) OR TX ("Externalising disorder") ) OR ( TI ( (Oppositional defiant disorder* or oppositional defiant problem*) ) OR AB ( (Oppositional defiant disorder* or oppositional defiant problem*) OR (MH"Attention Deficit and Disruptive Behavior Disorders+") ) OR ( TI ( (Psychos* or psychotic disorder* or psychotic problem*) ) OR AB ( (Psychos* or psychotic disorder* or psychotic problem*) ) OR MH ("Psychotic Disorders+) ) OR ( TI ( ( Anxiety or depression or depressive or "obsessive compulsive disorder" or "OCD" or phobia or phobic or mood disorder or anxiety disorder or panic disorder or agoraphobia or internalising problem* or internalising problem* or internalizing problem* or internalizing disorder* ) ) OR AB ( ( Anxiety or depression or depressive or "obsessive compulsive disorder" or "OCD" or phobia or phobic or mood disorder or anxiety disorder or panic disorder or agoraphobia or internalising problem* or internalising problem* or internalizing problem* or internalizing disorder* ) OR (MH "Depressive Disorder") OR (MH "Depressive Disorder, Major") OR (MH "Depressive Disorder, Treatment-Resistant") OR (MH "Dysthymic Disorder") OR (MM "Anxiety Disorders+") ) |
| S4 (Needs) | TI ("Information need*" or "knowledge need*" or need* or support or experience*or impact or wellbeing or concern* or want or perspective* or belief* or attitude*or prefer* or anxiety or anxious or depressed or depression or strain or stress or burden or "parent satisfaction" or "family relationship" or "parent* self-efficacy") OR AB ("Information need*" or "knowledge need*" or need* or support or experience*or impact or wellbeing or concern* or want or perspective* or belief* or attitude*or prefer* or anxiety or anxious or depressed or depression or strain or stress or burden or "parent satisfaction" or "family relationship" or "parent* self-efficacy")) |
| S5 | S1 N8 S4 |
| S6 | S2 N8 S3 |
| S7 | S5 AND S6 |

**Database:** PsycINFO

**Date:** 8th November 2022

| Search | Terms |
|---|---|
| S1 (Parent) | TI ((parent or parents or parental or mother or father or care*giver or guardian* or carer* or paternal or maternal ) ) OR AB ( ( parent or parents or parental or mother or father or care*giver or guardian* or carer* or paternal or maternal ) ) OR MM ("Parents+") |
| S2 (Children) | TI ( (children or adolescent* or adolescence or youth* or child or teenager* or pediatric* or paediatric* or kid* or teen* or young person or young people or boy* or girl* or juvenile* ) ) OR AB ( ( children or adolescent* or adolescence or youth* or child or teenager* or pediatric* or paediatric* or kid* or teen* or young person or young people or boy* or girl* or juvenile* ) ) OR MH ("Child+") OR MM ("Adolescent") |
| S3 (Mental Health) | (TI ( ( attention deficit disorder* or "attention deficit hyperactive disorder* "or "ADHD") OR AB (attention deficit disorder* or "attention deficit hyperactive disorder* "or "ADHD")OR MH ("Attention Deficit Disorder with Hyperactivity") OR ( TI ( (Eating disorder* or eating problem*) ) OR AB ( (Eating disorder* or eating problem*) OR MH("Feeding and Eating Disorders+") OR ( TI ( (Emerging personality disorder* or emerging personality problem*) ) OR AB ( (Emerging personality disorder* or emerging personality problem*) ) OR MH ("Personality Disorder+") ) OR ( TI ( (Externalising disorder* or externalising problem* or externalizing disorder* or externalizing problem*) OR AB (Externalising disorder* or externalising problem* or externalizing disorder* or externalizing problem*) OR TX ("Externalising disorder") ) OR ( TI ( (Oppositional defiant disorder* or oppositional defiant problem*) ) OR AB ( (Oppositional defiant disorder* or oppositional defiant problem*) OR (MH"Attention Deficit and Disruptive Behavior Disorders+") ) OR ( TI ( (Psychos* or psychotic disorder* or psychotic problem*) ) OR AB ( (Psychos* or psychotic disorder* or psychotic problem*) ) OR MH ("Psychotic Disorders+) ) OR ( TI ( ( Anxiety or depression or depressive or "obsessive compulsive disorder" or "OCD" or phobia or phobic or mood disorder or anxiety disorder or panic disorder or agoraphobia or internalising problem* or internalising problem* or internalizing problem* or internalizing disorder* ) ) OR AB ( ( Anxiety or depression or depressive or "obsessive compulsive disorder" or "OCD" or phobia or phobic or mood disorder or anxiety disorder or panic disorder or agoraphobia or internalising problem* or internalising problem* or internalizing problem* or internalizing disorder* ) OR (MH "Depressive Disorder") OR (MH "Depressive Disorder, Major") OR (MH "Depressive Disorder, Treatment-Resistant") OR (MH "Dysthymic Disorder") OR (MM "Anxiety Disorders+") ) |
| S4 (Needs) | TI ("Information need*" or "knowledge need*" or need* or support or experience*or impact or wellbeing or concern* or want or perspective* or belief* or attitude*or prefer* or anxiety or anxious or depressed or depression or strain or stress or burden or "parent satisfaction" or "family relationship" or "parent* self-efficacy") OR AB ("Information need*" or "knowledge need*" or need* or support or experience*or |

| | |
|---|---|
| | impact or wellbeing or concern* or want or perspective* or belief* or attitude*or prefer* or anxiety or anxious or depressed or depression or strain or stress or burden or "parent satisfaction" or "family relationship" or "parent* self-efficacy")) |
| S5 | S1 N8 S4 |
| S6 | S2 N8 S3 |
| S7 | S5 AND S6 |
| S8 | S5 AND S6 (English only) |

**Database:** CINAHL ULTIMATE

**Date:** 8th November 2022

| Search | Terms |
|---|---|
| S1 (Parent) | TI ((parent or parents or parental or mother or father or care*giver or guardian* or carer* or paternal or maternal ) ) OR AB ( ( parent or parents or parental or mother or father or care*giver or guardian* or carer* or paternal or maternal ) ) OR MM ("Parents+") |
| S2 (Children) | TI ( (children or adolescent* or adolescence or youth* or child or teenager* or pediatric* or paediatric* or kid* or teen* or young person or young people or boy* or girl* or juvenile* ) ) OR AB ( ( children or adolescent* or adolescence or youth* or child or teenager* or pediatric* or paediatric* or kid* or teen* or young person or young people or boy* or girl* or juvenile* ) ) OR MH ("Child+") OR MM ("Adolescent") |
| S3 (Mental Health) | ( TI ( ( attention deficit disorder* or "attention deficit hyperactive disorder* "or "ADHD") OR AB (attention deficit disorder* or "attention deficit hyperactive disorder* "or "ADHD")OR MH ("Attention Deficit Disorder with Hyperactivity") OR ( TI ( (Eating disorder* or eating problem*) ) OR AB ( (Eating disorder* or eating problem*) OR MH("Feeding and Eating Disorders+") OR ( TI ( (Emerging personality disorder* or emerging personality problem*) ) OR AB ( (Emerging personality disorder* or emerging personality problem*) ) OR MH ("Personality Disorder+") ) OR ( TI ( (Externalising disorder* or externalising problem* or externalizing disorder* or externalizing problem*) OR AB (Externalising disorder* or externalising problem* or externalizing disorder* or externalizing problem*) OR TX ("Externalising disorder") ) OR ( TI ( (Oppositional defiant disorder* or oppositional defiant problem*) ) OR AB ( (Oppositional defiant disorder* or oppositional defiant problem*) OR (MH"Attention Deficit and Disruptive Behavior Disorders+") ) OR ( TI ( (Psychos* or psychotic disorder* or psychotic problem*) ) OR AB ( (Psychos* or psychotic disorder* or psychotic problem*) ) OR MH ("Psychotic Disorders+) ) OR ( TI ( ( Anxiety or depression or depressive or "obsessive compulsive disorder" or "OCD" or phobia or phobic or mood disorder or anxiety disorder or panic disorder or agoraphobia or internalising problem* or internalising problem* or internalizing problem* or internalizing disorder* ) ) OR AB ( ( Anxiety or depression or depressive or "obsessive compulsive disorder" or "OCD" or phobia or phobic or mood disorder or anxiety disorder or panic disorder or agoraphobia or internalising problem* or internalising problem* or internalizing problem* or internalizing disorder* ) OR (MH "Depressive Disorder") OR (MH "Depressive Disorder, Major") OR (MH "Depressive Disorder, Treatment-Resistant") OR (MH "Dysthymic Disorder") OR (MM "Anxiety Disorders+") ) |
| S4 (Needs) | TI ("Information need*" or "knowledge need*" or need* or support or experience*or impact or wellbeing or concern* or want or perspective* or belief* or |

| | |
|---|---|
| | attitude*or prefer* or anxiety or anxious or depressed or depression or strain or stress or burden or “parent satisfaction” or “family relationship” or “parent* self-efficacy”) OR AB  (“Information need*” or “knowledge need*” or need* or support or experience*or impact or wellbeing or concern* or want or perspective* or belief* or attitude*or prefer* or anxiety or anxious or depressed or depression or strain or stress or burden or “parent satisfaction” or “family relationship” or “parent* self-efficacy”)) |
| S5 | S1 N8 S4 |
| S6 | S2 N8 S3 |
| S7 | S5 AND S6 |
| S8 | S5 AND S6 (English only) |

**Database:** AMED

**Date:** 8th November 2022

| **Search** | **Terms** |
|---|---|
| S1 (Parent) | TI ((parent or parents or parental or mother or father or care*giver or guardian* or carer* or paternal or maternal ) ) OR AB ( ( parent or parents or parental or mother or father or care*giver or guardian* or carer* or paternal or maternal ) ) OR MM ("Parents+") |
| S2 (Children) | TI ( (children or adolescent* or adolescence or youth* or child or teenager* or pediatric* or paediatric* or kid* or teen* or young person or young people or boy* or girl* or juvenile* ) ) OR AB ( ( children or adolescent* or adolescence or youth* or child or teenager* or pediatric* or paediatric* or kid* or teen* or young person or young people or boy* or girl* or juvenile* ) ) OR MH ("Child+") OR MM ("Adolescent") |
| S3 (Mental Health) | ( TI ( ( attention deficit disorder* or “attention deficit hyperactive disorder* “or “ADHD”) OR AB (attention deficit disorder* or “attention deficit hyperactive disorder* “or “ADHD”)OR MH (“Attention Deficit Disorder with Hyperactivity”) OR ( TI ( (Eating disorder* or eating problem*) ) OR AB ( (Eating disorder* or eating problem*) OR MH(“Feeding and Eating Disorders+”) OR ( TI ( (Emerging personality disorder* or emerging personality problem*) ) OR AB ( (Emerging personality disorder* or emerging personality problem*) ) OR MH (“Personality Disorder+”) ) OR ( TI ( (Externalising disorder* or externalising problem* or externalizing disorder* or externalizing problem*) OR AB (Externalising disorder* or externalising problem* or externalizing disorder* or externalizing problem*) OR TX (“Externalising disorder”) ) OR ( TI ( (Oppositional defiant disorder* or oppositional defiant problem*) ) OR AB ( (Oppositional defiant disorder* or oppositional defiant problem*) OR (MH“Attention Deficit and Disruptive Behavior Disorders+”) ) OR ( TI ( (Psychos* or psychotic disorder* or psychotic problem*) ) OR AB ( (Psychos* or psychotic disorder* or psychotic problem*) ) OR MH (“Psychotic Disorders+) ) OR ( TI ( ( Anxiety or depression or depressive or “obsessive compulsive disorder” or “OCD” or phobia or phobic or mood disorder or anxiety disorder or panic disorder or agoraphobia or internalising problem* or internalising problem* or internalizing problem* or internalizing disorder* ) ) OR AB ( ( Anxiety or depression or depressive or “obsessive compulsive disorder” or “OCD” or phobia or phobic or mood disorder or anxiety disorder or panic disorder or agoraphobia or internalising problem* or internalising problem* or internalizing problem* or internalizing disorder* ) OR (MH "Depressive Disorder") OR (MH "Depressive Disorder, Major") OR (MH "Depressive Disorder, Treatment-Resistant") OR (MH "Dysthymic Disorder") OR (MM "Anxiety Disorders+") ) |

For peer review only

| | |
|---|---|
| S4 (Needs) | TI ("Information need*" or "knowledge need*" or need* or support or experience*or impact or wellbeing or concern* or want or perspective* or belief* or attitude*or prefer* or anxiety or anxious or depressed or depression or strain or stress or burden or "parent satisfaction" or "family relationship" or "parent* self-efficacy") OR AB ("Information need*" or "knowledge need*" or need* or support or experience*or impact or wellbeing or concern* or want or perspective* or belief* or attitude*or prefer* or anxiety or anxious or depressed or depression or strain or stress or burden or "parent satisfaction" or "family relationship" or "parent* self-efficacy")) |
| S5 | S1 N8 S4 |
| S6 | S2 N8 S3 |
| S7 | S5 AND S6 |
| S8 | S5 AND S6 (English only) |

**Database:** The Cochrane Library (including the Cochrane Database of Systematic Reviews, the Cochrane Central Register of Controlled Trials (CENTRAL), the Database of Abstracts of Reviews of Effects, the Health Technology Assessment Database, and the NHS Economic Evaluation Database)

**Question number:** 1

**Date:** 8th November 2022

| Search | Terms |
|---|---|
| S1 (Parent) | TI ((parent or parents or parental or mother or father or care*giver or guardian* or carer* or paternal or maternal ) ) OR AB ( ( parent or parents or parental or mother or ather or care*giver or guardian* or carer* or paternal or maternal ) ) OR MM ("Parents+") |
| S2 (Children) | TI ( (children or adolescent* or adolescence or youth* or child or teenager* or pediatric* or paediatric* or kid* or teen* or young person or young people or boy* or girl* or juvenile* ) ) OR AB ( ( children or adolescent* or adolescence or youth* or child or teenager* or pediatric* or paediatric* or kid* or teen* or young person or young people or boy* or girl* or juvenile* ) ) OR MH ("Child+") OR MM ("Adolescent") |
| S3 (Mental Health)* | attention deficit disorder* or attention deficit hyperactive disorder* or ADHD or Eating disorder* or eating problem* or Emerging personality disorder or emerging personality problem* or Externalising disorder* or externalising problem* or externalizing disorder* or externalizing problem* or Oppositional defiant disorder* or oppositional defiant problem* or Psychos* or psychotic disorder* or psychotic problem* or Anxiety or depression or depressive or obsessive compulsive disorder or OCD or phobia or phobic or mood disorder or anxiety disorder or panic disorder or agoraphobia or internalising problem* or internalising problem* or internalizing problem* or internalizing disorder* |
| S4 (Needs) | TI ("Information need*" or "knowledge need*" or need* or support or experience*or impact or wellbeing or concern* or want or perspective* or belief* or attitude*or prefer*) OR AB ("Information need*" or "knowledge need*" or need* or support or experience*or impact or wellbeing or concern* or want or perspective* or belief* or attitude*or prefer*) or (Anxiety) or (depression) or (stress) or (burnout) or (parent satisfaction) or (family relationships) or (parenting self-efficacy) |
| S5 | S1 AND S2 AND S3 AND S4 |

For peer review only

**Database:** EMBASE

**Date:** 8th November 2022

| Search | Terms |
|---|---|
| S1 (Parent) | (parent or parents or parental or mother or father or care*giver or guardian* or carer* or paternal or maternal).ab. or (parent or parents or parental or mother or father or care*giver or guardian* or carer* or paternal or maternal).ti. or parents.kw. |
| S2 (Children) | (children or adolescent* or adolescence or youth* or child or teenager* or pediatric* or paediatric* or kid* or teen* or young person or young people or boy* or girl* or juvenile*).ab. or (children or adolescent* or adolescence or youth* or child or teenager* or pediatric* or paediatric* or kid* or teen* or young person or young people or boy* or girl* or juvenile*).ti. or (child or adolescent).kw. |
| S3 (Mental Health)* | (attention deficit disorder* or attention deficit hyperactive disorder* or ADHD or Eating disorder* or eating problem* or Emerging personality disorder or emerging personality problem* or Externalising disorder* or externalising problem* or externalizing disorder* or externalizing problem* or Oppositional defiant disorder* or oppositional defiant problem* or Psychos* or psychotic disorder* or psychotic problem* or Anxiety or depression or depressive or obsessive compulsive disorder or OCD or phobia or phobic or mood disorder or anxiety disorder or panic disorder or agoraphobia or internalising problem* or internalising problem* or internalizing problem* or internalizing disorder*).ab. or (attention deficit disorder* or attention deficit hyperactive disorder* or ADHD or Eating disorder* or eating problem* or Emerging personality disorder or emerging personality problem* or Externalising disorder* or externalising problem* or externalizing disorder* or externalizing problem* or Oppositional defiant disorder* or oppositional defiant problem* or Psychos* or psychotic disorder* or psychotic problem* or Anxiety or depression or depressive or obsessive compulsive disorder or OCD or phobia or phobic or mood disorder or anxiety disorder or panic disorder or agoraphobia or internalising problem* or internalising problem* or internalizing problem* or internalizing disorder*).ti. |
| S4 (Needs) | (information need* or knowledge need* or need* or support or experience*or impact or wellbeing or concern* or want or perspective* or belief* or attitude* or prefer* or anxiety or anxious or depressed or depression or strain or stress or burden or parent satisfaction or family relationship or parent* self-efficacy).ab. or (information need* or knowledge need* or need* or support or experience*or impact or wellbeing or concern* or want or perspective* or belief* or attitude* or prefer* or anxiety or anxious or depressed or depression or strain or stress or burden or parent satisfaction or family relationship or parent* self-efficacy).ti. |
| S5 | S1 adj8 s4 |
| S6 | S2 adj8 s5 |
| S7 | S5 and s6 |

**Database:** WoS

**Data conducted the search on** 14th November 2022

| Search | Terms |
|---|---|
| 1 | TS=(parent OR parents OR parental OR mother OR father OR care*giver OR guardian* OR carer* OR paternal OR maternal) |
| 2 | TS=(children OR adolescent* OR adolescence OR youth* OR child OR teenager* OR pediatric* OR paediatric* OR kid* OR teen* OR 'young person' OR 'young people' OR boy* OR girl* OR juvenile*) |

| | |
|---|---|
| 3 | TS=('attention deficit disorder*' OR 'attention deficit hyperactive disorder*' OR adhd OR 'attention deficit disorder with hyperactivity' OR 'eating disorder*' OR 'eating problem*' OR 'feeding and eating disorders' OR 'emerging personality disorder*' OR 'emerging personality problem*' OR 'personality disorder' OR 'externalising disorder*' OR 'externalising problem*' OR 'externalizing disorder*' OR 'externalizing problem*' OR 'oppositional defiant disorder*' OR 'oppositional defiant problem*' OR 'attention deficit and disruptive behavior disorders' OR psychos* OR 'psychotic disorder*' OR 'psychotic problem*' OR anxiety OR depression OR depressive OR 'obsessive compulsive disorder' OR 'ocd' OR phobia OR phobic OR 'mood disorder' OR 'anxiety disorder' OR 'panic disorder' OR agoraphobia OR 'internalising problem*' OR 'internalising disorder*' OR 'internalizing problem*' OR 'internalizing disorder*) |
| 4 | TS=("Information need*" or "knowledge need*" or need* or support or experience*or impact or wellbeing or concern* or want or perspective* or belief* or attitude*or prefer* or anxiety or anxious or depressed or depression or strain or stress or burden or "parent satisfaction" or "family relationship" or "parent* self-efficacy" ) |
| 5 | TS=((parent OR parents OR parental OR mother OR father OR care*giver OR guardian* OR carer* OR paternal OR maternal) NEAR/8 ("Information need*" or "knowledge need*" or support or experience* OR impact* or wellbeing or concern* or want or perspective* or belief* or attitude* or prefer* or anxiety or anxious or depressed or depression or strain or stress or burden or "parent satisfaction" or "family relationship" or "parent* self-efficacy")) |
| 6 | TS=(("attention deficit disorder" or "attention deficit hyperactive disorder" or adhd or "attention deficit disorder with hyperactivity" or "eating disorder" or "eating problem" or "feeding and eating disorder" or "emerging personality disorder" or "emerging personality problem" or "personality disorder" or "externalising disorder" or "externalising problem" or "oppositional defiant disorder" or "oppositional defiant problem" or "attention deficit and disruptive behavior disorders" or psychos* or "psychotic" or anxiety or depression or depressive or anxious or "obsessive compulsive disorder" or OCD or phobia or phobic or "mood disorder" or "anxiety disorder" or "panic disorder" or agoraphobia or "internalising problem" or "internalising disorder" or "internalizing problem" or "internalizing disorder" or "externalizing problem" or "externalizing disorder") NEAR/8 (children or adolescent* or adolescence or youth* or child or teenager* or pediatric* or paediatric* or kid* or teen* or "young person" or "young people" or boy* or girl* or juvenile*)) |
| 7 | #5 AND #6 |
| 8 | #7 English |

For peer review only

# PRISMA-P 2015 Checklist

**This checklist has been adapted for use with systematic review protocol submissions to BioMed Central journals from Table 3 in Moher D et al:** Preferred reporting items for systematic review and meta-analysis protocols (PRISMA-P) 2015 statement. *Systematic Reviews* 2015 **4**:1

An Editorial from the Editors-in-Chief of *Systematic Reviews* details why this checklist was adapted - **Moher D, Stewart L & Shekelle P:** Implementing PRISMA-P: recommendations for prospective authors. *Systematic Reviews* 2016 **5**:15

| Section/topic | # | Checklist item | Information reported | | Line number(s) |
|---|---|---|---|---|---|
| | | | Yes | No | |
| **ADMINISTRATIVE INFORMATION** | | | | | |
| **Title** | | | | | |
| Identification | 1a | Identify the report as a protocol of a systematic review | ✓ | | 1 |
| Update | 1b | If the protocol is for an update of a previous systematic review, identify as such | | ✓ | Not applicable |
| **Registration** | 2 | If registered, provide the name of the registry (e.g., PROSPERO) and registration number in the Abstract | | ✓ | Not applicable, NIHRA Funded |
| **Authors** | | | | | |
| Contact | 3a | Provide name, institutional affiliation, and e-mail address of all protocol authors; provide physical mailing address of corresponding author | ✓ | | 4-15 |
| Contributions | 3b | Describe contributions of protocol authors and identify the guarantor of the review | ✓ | | 233-2347 |
| **Amendments** | 4 | If the protocol represents an amendment of a previously completed or published protocol, identify as such and list changes; otherwise, state plan for documenting important protocol amendments | | ✓ | Not applicable |
| **Support** | | | | | |
| Sources | 5a | Indicate sources of financial or other support for the review | ✓ | | 241-243 |
| Sponsor | 5b | Provide name for the review funder and/or sponsor | ✓ | | 241-243 |
| Role of sponsor/funder | 5c | Describe roles of funder(s), sponsor(s), and/or institution(s), if any, in developing the protocol | | ✓ | Not applicable |
| **INTRODUCTION** | | | | | |
| **Rationale** | 6 | Describe the rationale for the review in the context of what is already known | ✓ | | 73-100 |
| **Objectives** | 7 | Provide an explicit statement of the question(s) the review will address with reference to participants, interventions, comparators, and outcomes (PICO) | ✓ | | 98-100 |
| **METHODS** | | | | | |

BioMed Central The Open Access Publisher



For peer review only

| Section/topic | # | Checklist item | Information reported Yes | No | Line number(s) |
|---|---|---|---|---|---|
| **Eligibility criteria** | 8 | Specify the study characteristics (e.g., PICO, study design, setting, time frame) and report characteristics (e.g., years considered, language, publication status) to be used as criteria for eligibility for the review | ✓ | | 121-134 |
| **Information sources** | 9 | Describe all intended information sources (e.g., electronic databases, contact with study authors, trial registers, or other grey literature sources) with planned dates of coverage | ✓ | | 146-155 |
| **Search strategy** | 10 | Present draft of search strategy to be used for at least one electronic database, including planned limits, such that it could be repeated | ✓ | | 146-163 |
| ***STUDY RECORDS*** | | | | | |
| Data management | 11a | Describe the mechanism(s) that will be used to manage records and data throughout the review | ✓ | | 166-172 |
| Selection process | 11b | State the process that will be used for selecting studies (e.g., two independent reviewers) through each phase of the review (i.e., screening, eligibility, and inclusion in meta-analysis) | ✓ | | 165-172 |
| Data collection process | 11c | Describe planned method of extracting data from reports (e.g., piloting forms, done independently, in duplicate), any processes for obtaining and confirming data from investigators | ✓ | | 173-189 |
| **Data items** | 12 | List and define all variables for which data will be sought (e.g., PICO items, funding sources), any pre-planned data assumptions and simplifications | ✓ | | 156-161 |
| **Outcomes and prioritization** | 13 | List and define all outcomes for which data will be sought, including prioritization of main and additional outcomes, with rationale | ✓ | | 174-187 |
| **Risk of bias in individual studies** | 14 | Describe anticipated methods for assessing risk of bias of individual studies, including whether this will be done at the outcome or study level, or both; state how this information will be used in data synthesis | ✓ | | 188-189 |
| ***DATA*** | | | | | |
| **Synthesis** | 15a | Describe criteria under which study data will be quantitatively synthesized | ✓ | | 190-211 |
| | 15b | If data are appropriate for quantitative synthesis, describe planned summary measures, methods of handling data, and methods of combining data from studies, including any planned exploration of consistency (e.g., $I^2$, Kendall's tau) | ✓ | | 190-211 |
| | 15c | Describe any proposed additional analyses (e.g., sensitivity or subgroup analyses, meta-regression) | ✓ | | 190-211 |
| | 15d | If quantitative synthesis is not appropriate, describe the type of summary planned | ✓ | | 211 |
| **Meta-bias(es)** | 16 | Specify any planned assessment of meta-bias(es) (e.g., publication bias across studies, selective reporting within studies) | ✓ | | 188-189 |
| **Confidence in cumulative evidence** | 17 | Describe how the strength of the body of evidence will be assessed (e.g., GRADE) | ✓ | | 188-189 |

For peer review only



BioMed Central The Open Access Publisher
