# Supplementary material for: Needs of parents and carers of children and young people with mental health difficulties: protocol for a systematic review
Source: BMJ Open. 2023 Feb 16;13(2):e071341. doi: 10.1136/bmjopen-2022-071341 (PMC9936057; doi:10.1136/bmjopen-2022-071341)
Supplement: Reviewer comments [file bmjopen-2022-071341.reviewer_comments.pdf]

PEER REVIEW HISTORY

BMJ Open publishes all reviews undertaken for accepted manuscripts. Reviewers are asked to complete a checklist review form (**http://bmjopen.bmj.com/site/about/resources/checklist.pdf**) and are provided with free text boxes to elaborate on their assessment. These free text comments are reproduced below.

ARTICLE DETAILS

**TITLE (PROVISIONAL)**	Needs of parents and carers of children and young people with mental health difficulties: protocol for a systematic review
**AUTHORS**	Martin, Faith; Dahmash, Dania; Glover, Sarah; Duncan, Charlie; Turner, Andy; Halligan, Sarah

This article was not externally reviewed at BMJ Open. Protocols that have been independently assessed prior to submission to BMJ Open are usually fast-tracked to publication on the grounds that further substantial changes will not be possible. This independent assessment will usually be external, independent review for both a substantial grant award from a non-commercial or government funder and ethics approval.
